# Adherence to four dietary indices and the risk of all‐cause and cause‐specific dementia: Findings from the UK Biobank study

**DOI:** 10.1111/dom.16609

**Published:** 2025-07-16

**Authors:** Fernanda Carrasco‐Marín, Solange Parra‐Soto, Marion Guerrero‐Wyss, Carolina Araya‐Bastias, Jirapitcha Boonpor, Carla Villagrán‐Cerro, Fanny Petermann‐Rocha, Oliver M. Shannon, John C. Mathers, Katherine M. Livingstone, Sara Dingle, Sofía Gálvez‐Tejeda, Ana Hernández‐Peregrina, Nishka Pranay, Guillermo Molina‐Recio, Jill Pell, Frederick Ho, Carlos Celis‐Morales, Rafael Molina‐Luque

**Affiliations:** ^1^ School of Cardiovascular and Metabolic Health University of Glasgow Glasgow UK; ^2^ Departamento de Enfermería, Farmacología y Fisioterapia, Facultad de Medicina y Enfermería Universidad de Córdoba Córdoba Spain; ^3^ Centro de Vida Saludable Universidad de Concepción Concepción Chile; ^4^ Department of Nutrition and Public Health, Faculty of Health Science and Food Universidad del Bío‐Bío Chillán Chile; ^5^ Faculty of Public Health Kasetsart University Sakon Nakhon Thailand; ^6^ Centro de Investigación Biomédica, Facultad de Medicina Universidad Diego Portales Santiago Chile; ^7^ Human Nutrition & Exercise Research Centre Centre for Healthier Lives, Population Health Sciences Institute, Newcastle University Newcastle UK; ^8^ Institute for Physical Activity and Nutrition School of Exercise and Nutrition Sciences, Deakin University Burwood Victoria Australia; ^9^ Lifestyles, Innovation and Health (GA‐16), Maimonides Biomedical Research Institute of Cordoba (IMIBIC) Córdoba Spain; ^10^ School of Health and Wellbeing University of Glasgow Glasgow UK; ^11^ Human Performance Lab, Education, Physical Activity and Health Research Unit University Católica del Maule Talca Chile; ^12^ Centro de Investigación en Medicina de Altura (CEIMA) Universidad Arturo Prat Iquique Chile

**Keywords:** Alzheimer disease, dementia, diet, vascular dementia

## Abstract

**Aims:**

Despite several modifiable risk factors for dementia being identified, diet is often excluded from consideration due to insufficient evidence. Although various healthy dietary indices have been associated with improved health outcomes, their link to dementia risk remains unclear. This study investigates the association between four dietary indices and dementia risk among UK Biobank participants.

**Materials and Methods:**

This study utilized data from the UK Biobank cohort. Dietary intake was self‐reported using repeated 24‐h recalls (up to five occasions), averaged for analysis. Adherence to a healthy diet was assessed using four dietary indices: the Recommended Food Score (RFS), Healthy Diet Indicator (HDI), Mediterranean Diet Score (MDS) and the Mediterranean‐DASH Intervention for Neurodegenerative Delay (MIND) score. The primary outcome was incident all‐cause dementia, Alzheimer disease, vascular dementia, and non‐vascular dementia extracted from hospital and primary care records using ICD‐10 codes. Censored Cox proportional hazards models were used to estimate the risk of developing dementia and reported as hazard ratios (HRs) with their 95% confidence interval (CI). Analyses were adjusted for age, sex, education and various lifestyle and health‐related factors.

**Results:**

The analysis included 121 521 participants (mean age: 55.7 years; 53.5% women), followed for a median of 10.9 years. During follow‐up, 621 participants developed dementia. Compared with individuals with the lowest dietary adherence (Quartile 1), those in the highest adherence group (Quartile 4) showed a lower risk of all‐cause dementia for MDS (HR: 0.53; 95% CI: 0.45–0.63), MIND (HR: 0.61; 95% CI: 0.48–0.78), and RFS (HR: 0.66; 95% CI: 0.53–0.85). Similar associations were observed for Alzheimer disease and MDS (HR: 0.55; 95% CI: 0.46–0.67), RFS (HR: 0.61; 95% CI: 0.47–0.80), and MIND (HR: 0.66; 95% CI: 0.51–0.87). For vascular dementia, only MDS (HR: 0.46; 95% CI: 0.31–0.68) and MIND (HR: 0.68; 95% CI: 0.40–0.97) showed a lower risk. These associations remained in a 5‐year landmark analysis. No associations were observed for HDI with any dementia outcome. Our findings indicate that higher adherence to specific dietary patterns, particularly the Mediterranean, RFS and MIND diet, is associated with a lower risk of dementia.

**Conclusions:**

The Mediterranean Diet demonstrated the strongest association. These results highlight the potential of healthy dietary patterns to improve brain health in older adults.

## 1 INTRODUCTION

As global life expectancy increases, so does the prevalence of dementia, characterized by progressive cognitive decline that significantly impacts the quality of life for affected individuals and their communities.^1^ Currently, over 55 million people worldwide are living with dementia, with projections indicating this number will surge to 139 million by 2050.^2^ Dementia arises from a complex interplay of genetic, environmental and lifestyle risk factors. While a definitive cure remains elusive, modifiable lifestyle factors have been highlighted as key elements for preventing or delaying the onset of dementia.^3^, ^4^

Among these factors, diet is notably absent from the most recent Dementia Expert Report as a recognized modifiable risk factor.^5^ The 2024 Lancet commission report highlights that, although numerous studies have explored the relationship between diet and dementia, findings remain inconsistent, particularly due to the negative results of some interventional trials, which have shown no significant cognitive benefits from specific dietary interventions. Much of the existing evidence also stems from small cohort studies or focuses on isolated nutrients or food groups rather than comprehensive dietary patterns that more accurately capture real‐world eating behaviours.^6^, ^7^, ^8^ Understanding dietary impacts on dementia risk requires assessing broader dietary quality, which can be captured through dietary quality indices.^7^ These indices evaluate adherence to established nutritional guidelines and dietary patterns, considering not just individual foods or nutrients but their combined effects on health.

Dietary indices such as the Recommended Food Score (RFS), Healthy Diet Indicator (HDI), Mediterranean Diet Score (MDS), and the Mediterranean‐DASH Intervention for Neurodegenerative Delay (MIND) diet provide a structured approach to measure dietary quality. The RFS emphasizes the intake of fruits, vegetables, whole grains and low‐fat dairy products, aligning with general dietary guidelines for health.^9^ The HDI, based on WHO recommendations, evaluates both food‐based and nutrient‐based elements, promoting polyunsaturated fats, high fibre, and limitating saturated fats and red meat.^10^ The MDS reflects traditional Mediterranean dietary patterns, prioritizing olive oil, fish, and moderate alcohol consumption, especially red wine.^9^ While red wine is often highlighted for its potential benefits due to its high antioxidant content, these claims should be interpreted with caution. Recent studies have raised concerns about potential selection bias and confounding factors that may influence these findings.^11^ Finally, the MIND diet combines aspects of the Mediterranean and DASH diets, emphasizing brain‐healthy foods like leafy greens and berries while restricting unhealthy fats and sugars.^12^

Recent studies using UK Biobank data, such as the one by Zhong et al. (2023),^13^ have reported associations between individual dietary components and dementia risk. However, these studies generally focused on single dietary exposures or specific diet scores, without comparing multiple indices within the same population or applying more conservative methodological strategies to minimize bias.

Therefore, this study aimed to fill key gaps by comparing the associations of these four dietary indices with dementia outcomes, including specific types such as Alzheimer disease and vascular dementia. Importantly, we extended the current understanding by conducting comprehensive adjustments for confounding variables and employing conservative landmark analysis to mitigate reverse causation, a common limitation in existing research.^14^ By evaluating and comparing these indices within the same population, we aim to provide a more robust and nuanced understanding of the role of diet in dementia prevention.

## 2 METHODS

Over half a million participants (aged 37–73 years) were enrolled in UK Biobank from the general population in England, Wales and Scotland via 22 assessment centres. All participants gave informed written consent to data collection via a touch‐screen questionnaire, including physical measurements and biological samples. A detailed description of the protocol and baseline assessments is available (https://www.ukbiobank.ac.uk/). For this study, 121 521 participants with data available for diet, dementia and covariates were included (Figure S1).

### 2.1 Incidence of dementia

Participants with dementia or any neurodegenerative condition at recruitment were excluded. Incident dementia (non‐fatal) was ascertained from hospital episodes and primary care records. Hospital admissions were identified via record linkage to Health Episode Statistics (HES) (England and Wales), and the Scottish Morbidity Records (Scotland). Details of the linkage procedure can be found at http://content.digital.nhs.uk/services. Hospital admissions data were available from 1997 to October 2022 in England, 1996 to August 2022 in Scotland, and from 1999 to March 2022 in Wales. Therefore, follow‐up was censored at these dates. Using the International Classification of Diseases, 10th revision (ICD‐10), Alzheimer disease and vascular dementia were defined using ICD‐10 codes: Alzheimer disease; vascular dementia F01; and all‐cause dementia (hereafter dementia). Non‐vascular dementia includes Alzheimer disease, as well as other neurodegenerative disorders like frontotemporal dementia and dementia with Lewy bodies.

### 2.2 Dietary indices

Dietary intake was assessed using the Oxford WebQ, a web‐based 24‐h dietary assessment tool.^15^ Energy and nutrient intake were calculated using McCance and Widdowson's ‘The Composition of Food’, 5th edition.^16^ Data was collected based on the previous day's intake with questions like: ‘Did you consume any of these foods yesterday?’ To determine nutrient intake, consumption frequency was multiplied by standard portion size and nutrient composition.^17^ The average of multiple 24‐h recalls was used, collected up to five occasions between April 2009 and June 2012, as detailed on the UK Biobank webpage (https://biobank.ndph.ox.ac.uk).

**FIGURE 1:**
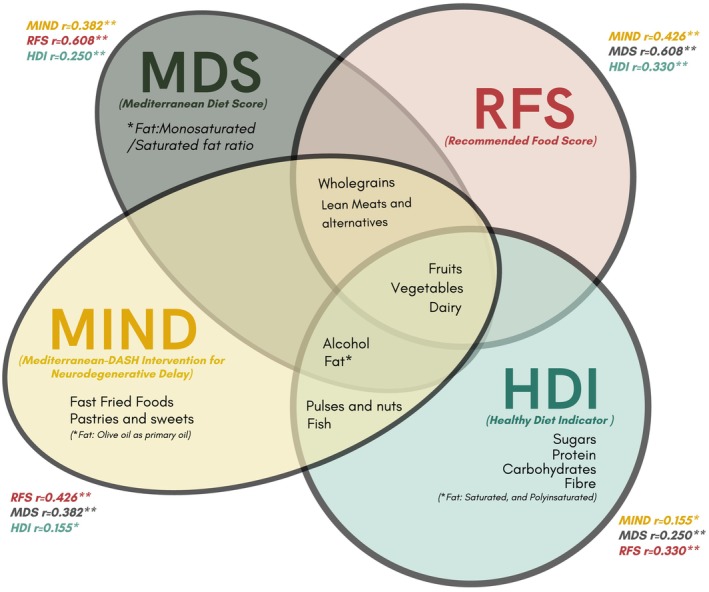
Correlation and overlap among dietary indices. Correlation coefficients between dietary indices are labelled as ‘*r*’. Correlation *p*‐values are indicated as *p < 0.001 and **p < 0.0001.

This study compared four dietary indices, each employing distinct methodologies for assessing diet quality. Brief descriptions of each index and specific foods or nutrients included are in Figure 1.

#### 2.2.1 Recommended Food Score (RFS)

The RFS is a food‐based index evaluating consumption of food groups aligned with dietary guidelines. Following the methodology of Livingstone et al. in 2021,^9^ five food groups are defined: fruits (seven items), vegetables (seven items), whole grains (two items), meat and alternatives (three items), and reduced‐fat dairy products (two items). In this system, 1 point is allocated when the consumption exceeds minimum thresholds of 15 g/day for non‐beverages, and 30 g/day for beverages. Intake below these thresholds scores 0. Scores ranged from 0 to 21, with higher scores indicating greater consumption of recommended foods.^9^

#### 2.2.2 Healthy Diet Indicator (HDI)

The HDI assesses diet quality based on World Health Organization (WHO) guidelines,^10^ considering food and nutrient‐based factors. An adapted version of the HDI by Livingstone et al.,^9^ consists of an 11‐point scale. The index includes saturated fat, polyunsaturated fat, protein, total carbohydrates, dietary fibre, fruits and vegetables, pulses and nuts, total non‐milk extrinsic sugars, fish, red meat and meat products, and calcium. Intake within the specified cut‐offs scores 1, while intake outside scores 0. Scores range from 0 to 11, with a higher score indicating a higher diet quality.^9^, ^18^

#### 2.2.3 Mediterranean Diet Score (MDS)

The MDS combines food and nutrient‐based components to assess adherence to a Mediterranean diet. We used a 9‐item index adapted by Livingstone et al. evaluating nine components: vegetables, legumes, fruits and nuts, cereals, fish and seafood, monounsaturated to saturated fats, dairy products, meat and meat products, and alcohol. Sex‐specific median intakes were used as cut‐off points. A score of 1 was assigned for above‐median intake of vegetables, legumes, fruits and nuts, cereals, fish and seafood; below‐median intake of meat, meat products and dairy products; and above median ratio of monounsaturated to saturated fat. Low to moderate alcohol intake (<14 unit/week) scored 1, while no intake or >14 unit/week scored 0. Total score ranged from 0 to 9, with higher scores indicating greater adherence.

#### 2.2.4 Mediterranean‐DASH Intervention for Neurodegenerative Delay (MIND)

MIND—described by Morris et al.—is a hybrid of the Mediterranean and DASH (dietary approaches to stop hypertension) diets.^12^ This comprises 15 components: 10 brain‐healthy food categories (leafy green vegetables, other vegetables, nuts, berries, legumes, whole grains, fish, poultry, olive oil, and wine) and five categories of unhealthy foods (red meats, stick butter and margarine, cheese, pastries and sweets, and fried/fast food). Scores range from 0 to 15, with 1 point for meeting recommended intake and 0 for not meeting it. For oil consumption, 1 point was assigned if olive oil was used in cooking; otherwise, 0 points.^19^

Each dietary index score was categorized into quartiles based on its distribution, as there were no predefined or standardized cut‐off points to distinguish between low and high adherence. Participants were therefore grouped into four adherence categories: lowest adherence (reference group), moderately low adherence, moderately high adherence and highest adherence. Cut‐off points for each of the scores are presented in Table S1.

### 2.3 Covariates

Covariates were selected from existing evidence on risk factors for dementia risk extracted from the Lancet Dementia report.^5^ Age was calculated using the date of birth and baseline assessment date. Ethnicity was self‐reported and categorized as Caucasian, South Asian, African descent, Chinese and mixed, or other. Educational attainment was self‐reported and categorized ordinally, following the International Standard Classification of Education^20^: College or University degree; pre‐university qualifications; O‐levels/GCSEs or equivalent; CSEs or equivalent; and None of the above.

Smoking status was categorized as never, former, or current smoker. Alcohol consumption was categorized as high ‘risk’ (>14 unit/week) or ‘no risk’ (<14 unit/week).^21^ Physical activity was assessed using the International Physical Activity Questionnaire, estimating total weekly metabolic equivalent of task (MET)^22^ and categorized as inactive, moderately active, or active. Social interaction was evaluated via frequency of family visits and engagement in activities like sports clubs or gyms, or religious groups.

Body mass index (BMI) was calculated using weight and height, and categorized per WHO criteria: underweight (<18.5 kg/m^2^), normal weight (18.5–24.9 kg/m^2^), overweight (25.0–29.9 kg/m^2^), and obese (≥30.0 kg/m^2^).^23^ Medical history, including physician‐diagnosed conditions such as diabetes, depression, hypertension, traumatic brain injury and hearing problems, along with cardiovascular medications, was self‐reported. Low‐Density Lipoprotein cholesterol (LDL) concentrations were measured using serum samples, and PM2.5 air pollution levels were estimated for participants' addresses using a Land Use Regression (LUR) model.

### 2.4 Statistical analyses

Baseline characteristics across quartiles of adherence to dietary indices were described using means (SDs) for continuous variables and frequencies (%) for categorical variables. Pearson correlation assessed within‐pattern correlations.

Associations between dietary indices and dementia outcomes—including all‐cause dementia, vascular dementia, non‐vascular dementia and Alzheimer disease—were assessed using Cox proportional hazards models, with follow‐up time as the underlying time scale. The proportional hazards assumption was evaluated using Schoenfeld residuals. Participants in the lowest quartile of dietary adherence served as the reference group. Linear trends across quartiles of adherence were examined by modelling adherence as an ordinal variable. Hazard ratios (HRs) and corresponding 95% confidence intervals (CIs) were calculated for each one‐quartile increase in adherence to each dietary index.

Participants with missing data on the dietary index (*n* = 380 999) or covariates (*n* = 2364) were excluded from the analysis (see Figure S1). The study included only those with complete data, following a complete case analysis approach. No imputation was performed for missing values. To address potential reverse causality, we conducted a 5‐year landmark analysis, excluding participants who experienced events within the first 5 years of follow‐up (*n* = 56).

Analyses were adjusted incrementally^5^: Model 0 (unadjusted); Model 1 (sociodemographic factors: age, sex, education and ethnicity); Model 2 (lifestyle: smoking, alcohol, physical activity, social interaction and PM2.5); and Model 3 was additionally adjusted for health‐related factors (BMI, hypertension, diabetes, depression, history of traumatic brain injury, hearing problems, LDL cholesterol and cardiovascular medications). Multicollinearity within covariates was assessed using the Variance Inflation Factor.

All analyses used Stata version 18, with *p* < 0.05 considered significant.

## 3 RESULTS

This study included 121 521 participants with complete data (Figure S1). The median follow‐up was 10.9 years (interquartile range: 10.3–11.5 years). During this period, 621 participants (0.51%) were diagnosed with dementia, 118 (0.10%) with vascular dementia, 503 (0.41%) with non‐vascular dementia and 260 (0.21%) with Alzheimer disease.

Table 1 presents the characteristics of the cohort, grouped by quartiles of adherence to the Mediterranean diet. Participants with higher adherence were more likely to be women of White European background and to have higher education levels compared with those in the lowest adherence quartile. Regarding lifestyle factors, these participants were also more likely to be never smokers, more physically active, report higher alcohol consumption, and have a slightly lower prevalence of long‐term conditions. Detailed cohort characteristics by quartiles of MIND, RFS and HDI dietary indices are provided in Tables S2–S4, respectively. Figure 1 presents the correlations between dietary indices, with magnitudes ranging from 0.155 to 0.608. The strongest correlation was observed between the Mediterranean diet and the RFS diet score, and the weakest was observed between MIND and HDI diet scores.

**TABLE 1:** Cohort characteristics by levels of adherence to the Mediterranean Diet Score (MDS).

	Lowest adherence	Moderately low adherence	Moderately high adherence	Highest adherence	Total
Total *n*	6556 (5.4%)	42 912 (35.3%)	27 697 (22.8%)	44 356 (36.5%)	121 521 (100.0%)
Age (years)	55.03 (8.20)	55.50 (8.16)	55.87 (7.94)	56.14 (7.78)	55.79 (7.98)
Sex					
Women	3415 (52.1%)	22 308 (52.0%)	13 775 (49.7%)	25 459 (57.4%)	64 957 (53.5%)
Men	3141 (47.9%)	20 604 (48.0%)	13 922 (50.3%)	18 897 (42.6%)	56 564 (46.5%)
Ethnicity					
White	6148 (93.8%)	40 972 (95.5%)	26 782 (96.7%)	42 917 (96.8%)	116 819 (96.1%)
Mixed	95 (1.4%)	566 (1.3%)	296 (1.1%)	572 (1.3%)	1529 (1.3%)
South Asian	203 (3.1%)	762 (1.8%)	308 (1.1%)	378 (0.9%)	1651 (1.4%)
Black	100 (1.5%)	520 (1.2%)	237 (0.9%)	362 (0.8%)	1219 (1.0%)
Chinese	10 (0.2%)	92 (0.2%)	74 (0.3%)	127 (0.3%)	303 (0.2%)
Education					
None of the above	843 (12.9%)	4304 (10.0%)	2108 (7.6%)	2600 (5.9%)	9855 (8.1%)
CSEs	455 (6.9%)	2361 (5.5%)	1147 (4.1%)	1360 (3.1%)	5323 (4.4%)
O‐levels	1697 (25.9%)	10 782 (25.1%)	6274 (22.7%)	8988 (20.3%)	27 741 (22.8%)
A‐levels	903 (13.8%)	6334 (14.8%)	4160 (15.0%)	6572 (14.8%)	17 969 (14.8%)
College/University degree	2658 (40.5%)	19 131 (44.6%)	14 008 (50.6%)	24 836 (56.0%)	60 633 (49.9%)
Smoking					
Never	3962 (60.5%)	24 972 (58.3%)	15 513 (56.1%)	24 228 (54.7%)	68 675 (56.6%)
Previous	2042 (31.2%)	14 374 (33.6%)	10 051 (36.3%)	17 242 (38.9%)	43 709 (36.0%)
Current	541 (8.3%)	3495 (8.2%)	2097 (7.6%)	2828 (6.4%)	8961 (7.4%)
Alcohol consumption (units/week)	9.12 (13.31)	13.89 (16.62)	17.38 (17.69)	18.92 (17.51)	16.26 (17.26)
Physical activity					
Inactive	1336 (20.4%)	8449 (19.7%)	5078 (18.3%)	6952 (15.7%)	21 815 (18.0%)
Moderate active	2731 (41.7%)	18 129 (42.2%)	11 939 (43.1%)	19 033 (42.9%)	51 832 (42.7%)
Active	2489 (38.0%)	16 334 (38.1%)	10 680 (38.6%)	18 371 (41.4%)	47 874 (39.4%)
Family/friends visits					
Almost daily	631 (9.6%)	4180 (9.7%)	2476 (8.9%)	3966 (8.9%)	11 253 (9.3%)
2–4 times a week	1906 (29.1%)	12 597 (29.4%)	8136 (29.4%)	13 129 (29.6%)	35 768 (29.4%)
About once a week	2368 (36.1%)	16 188 (37.7%)	10 252 (37.0%)	16 565 (37.3%)	45 373 (37.3%)
About once a month	1010 (15.4%)	6431 (15.0%)	4416 (15.9%)	7113 (16.0%)	18 970 (15.6%)
Once every few months	530 (8.1%)	2930 (6.8%)	2057 (7.4%)	3071 (6.9%)	8588 (7.1%)
Never or almost never	103 (1.6%)	521 (1.2%)	328 (1.2%)	456 (1.0%)	1408 (1.2%)
No friends/family	8 (0.1%)	65 (0.2%)	32 (0.1%)	56 (0.1%)	161 (0.1%)
Leisure social activities					
None of the above	2190 (33.4%)	12 731 (29.7%)	7588 (27.4%)	11 486 (25.9%)	33 995 (28.0%)
Sports club or gym	1967 (30.0%)	14 058 (32.8%)	9642 (34.8%)	16 666 (37.6%)	42 333 (34.8%)
Pub or social club	877 (13.4%)	6696 (15.6%)	4536 (16.4%)	6902 (15.6%)	19 011 (15.6%)
Religious group	707 (10.8%)	4287 (10.0%)	2518 (9.1%)	3554 (8.0%)	11 066 (9.1%)
Adult education class	194 (3.0%)	1218 (2.8%)	887 (3.2%)	1672 (3.8%)	3971 (3.3%)
Other group activity	621 (9.5%)	3922 (9.1%)	2526 (9.1%)	4076 (9.2%)	11 145 (9.2%)
Air pollution, PM 2.5 (μg/m^3^)	9.93 (1.00)	9.90 (1.01)	9.90 (1.04)	9.92 (1.05)	9.91 (1.03)
Body mass index (kg m^2^)	27.06 (4.70)	26.96 (4.60)	26.75 (4.43)	26.41 (4.42)	26.72 (4.51)
CVD medication					
No	5567 (84.9%)	36 228 (84.4%)	23 229 (83.9%)	37 396 (84.3%)	102 420 (84.3%)
Yes	989 (15.1%)	6684 (15.6%)	4468 (16.1%)	6960 (15.7%)	19 101 (15.7%)
Type 2 diabetes					
No	6260 (95.5%)	41 148 (95.9%)	26 626 (96.1%)	42 833 (96.6%)	116 867 (96.2%)
Yes	296 (4.5%)	1764 (4.1%)	1071 (3.9%)	1523 (3.4%)	4654 (3.8%)
Hypertension					
No	5120 (78.1%)	33 158 (77.3%)	21 384 (77.2%)	34 330 (77.4%)	93 992 (77.3%)
Yes	1436 (21.9%)	9754 (22.7%)	6313 (22.8%)	10 026 (22.6%)	27 529 (22.7%)
Depression					
No	6176 (94.2%)	40 610 (94.6%)	26 419 (95.4%)	42 381 (95.5%)	115 586 (95.1%)
Yes	380 (5.8%)	2302 (5.4%)	1278 (4.6%)	1975 (4.5%)	5935 (4.9%)
LDL cholesterol (mmol/L)	3.53 (0.84)	3.56 (0.85)	3.55 (0.85)	3.55 (0.84)	3.55 (0.85)
Traumatic brain injury					
No	6528 (99.6%)	42 699 (99.5%)	27 577 (99.6%)	44 152 (99.5%)	120 956 (99.5%)
Yes	28 (0.4%)	213 (0.5%)	120 (0.4%)	204 (0.5%)	565 (0.5%)
Hearing problems					
No	4913 (74.9%)	31 876 (74.3%)	20 579 (74.3%)	33 473 (75.5%)	90 841 (74.8%)
Yes	1641 (25.0%)	11 024 (25.7%)	7112 (25.7%)	10 878 (24.5%)	30 655 (25.2%)

*Note*: Data presented as mean and standard deviation for continuous variables and as frequency and % for categorical variables.

Abbreviations: BMI, body mass index; MDS, Mediterranean Diet Score; IPAQ, International Physical Activity Questionnaire.

**FIGURE 2:**
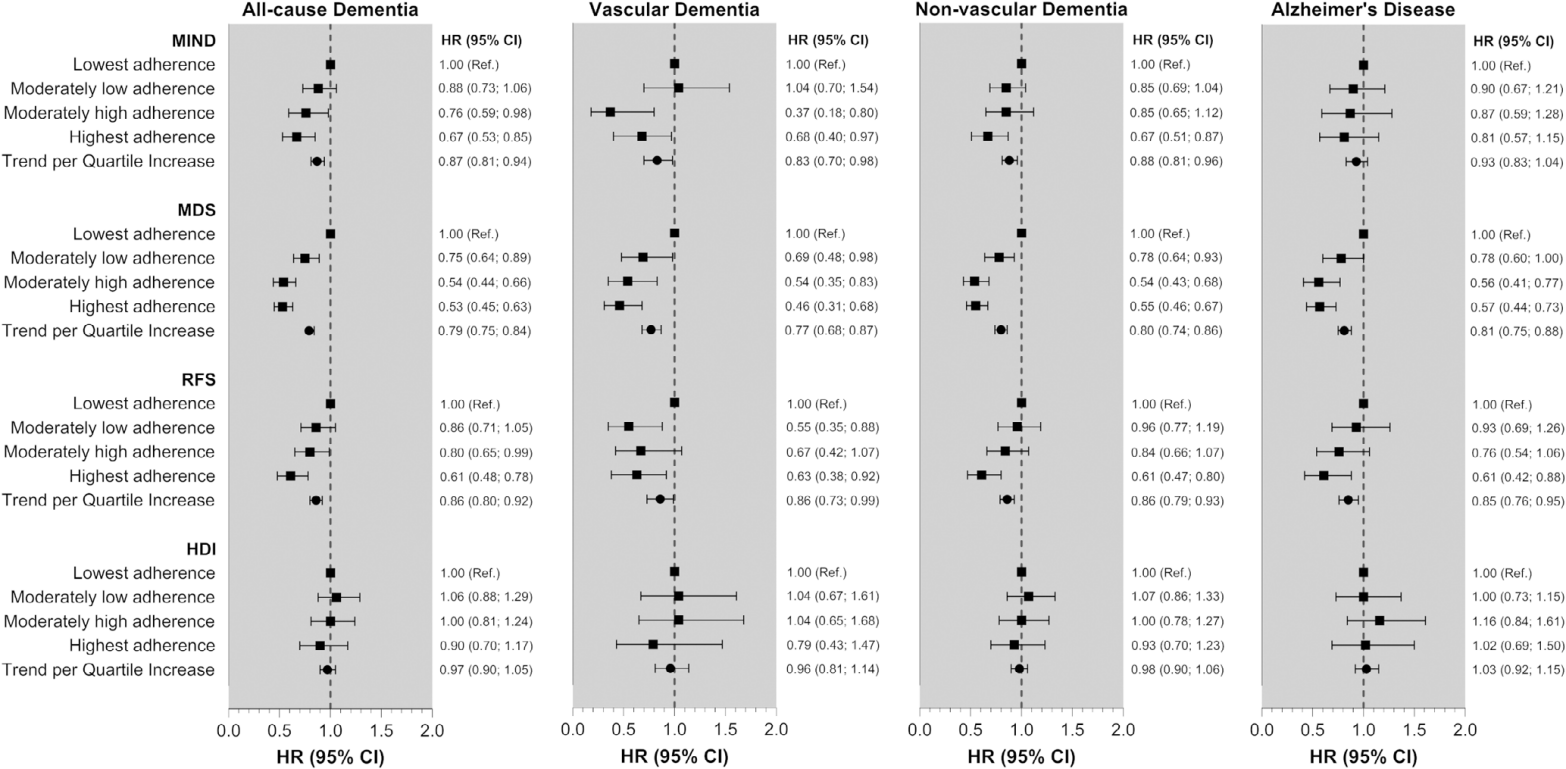
Associations of levels of adherence to dietary indices with all‐cause and specific cause dementia incidence. Data are presented as adjusted hazard ratios (HRs) with 95% confidence intervals (CIs) per quartiles of adherence to each of the four dietary indices. Low adherence to the diet index (lowest adherence, quartile 1) was used as the reference group. The trend represents the risk of dementia associated with each 1‐quartile increase in adherence. Analyses were adjusted for sociodemographic factors (age, sex, education and ethnicity), lifestyle‐related factors (smoking, alcohol intake, physical activity, social interaction and PM2.5 exposure), and health‐related factors (BMI, hypertension, diabetes, depression, history of traumatic brain injury, hearing problems, LDL cholesterol, cardiovascular medications and multimorbidity).

Figure 2 shows the associations between the four dietary indices and the risk of dementia and its subtypes. In our most adjusted model (model 3), an inverse association was observed between adherence to the Mediterranean, MIND, and RFS dietary scores, but not the HDI, and the risk of all‐cause dementia. Compared with individuals in the lowest adherence quartile, those in the highest adherence quartile had a lower risk of all‐cause dementia: MDS (HR: 0.53; 95% CI: 0.45, 0.63) MIND (HR: 0.67; 95% CI: 0.53, 0.85) and RFS (HR: 0.61; 95% CI: 0.48, 0.78) (Table S5).

Similar results were found for vascular dementia; participants with higher adherence to the Mediterranean diet had a lower risk (HR: 0.46; 95% CI: 0.31, 0.68), and those with high adherence to the MIND diet also showed a reduced risk (HR:0.68; 95% CI: 0.40, 0.97). The association between the RFS diet and vascular dementia was borderline significant (HR: 0.63; 95% CI: 0.38, 1.04) (Figure 2 and Table S6). For non‐vascular dementia, higher adherence to the Mediterranean (HR: 0.55; 95% CI: 0.46: 0.67), MIND (HR: 0.67; 95% CI: 0.51, 0.87), and RFS (HR:0.61; 95% CI: 0.47, 0.80) diets were associated with lower risk (Figure 2 and Table S7).

For Alzheimer disease, only the Mediterranean (HR:0.57; 95% CI: 0.44, 0.73) and RFS (HR:0.61; 95% CI: 0.42, 0.88) scores were associated with a reduced risk for participants in the highest adherence quartile compared with those in the lowest (Figure 2 and Table S8). Associations for other models with different levels of adjustment are detailed in Tables S5–S8.

**FIGURE 3:**
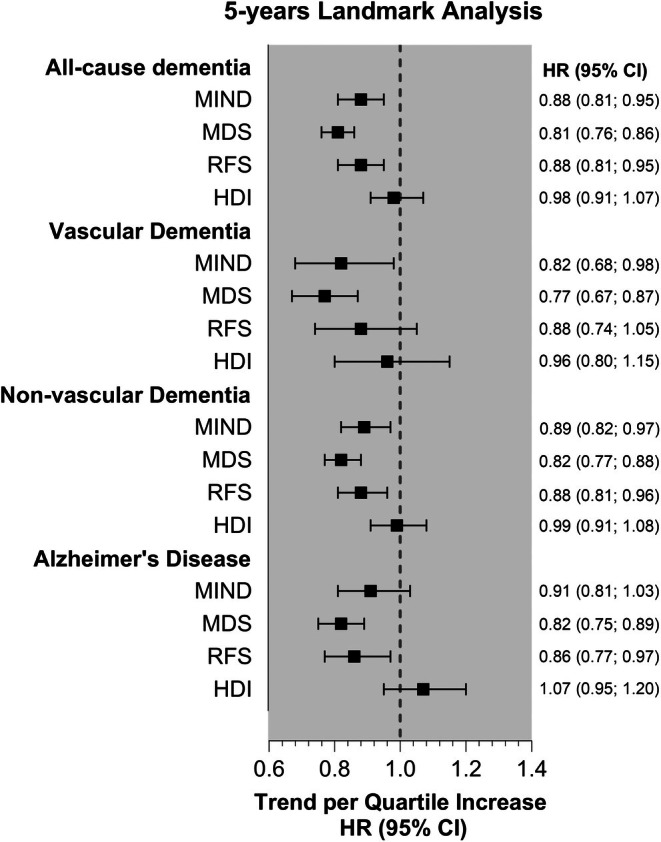
Associations of adherence to dietary indices with all‐cause and cause‐specific dementia incidence using a 5‐year landmark analysis. Data are presented as adjusted hazard ratios (HRs) with 95% confidence intervals (CIs) per quartile increase in adherence to the dietary pattern (Trend HR). Analyses were adjusted for sociodemographic factors (age, sex, education and ethnicity), lifestyle‐related factors (smoking, alcohol intake, physical activity, social interaction and PM2.5 exposure), and health‐related factors (BMI, hypertension, diabetes, depression, history of traumatic brain injury, hearing problems, LDL cholesterol, cardiovascular medications and multimorbidity). A 5‐year landmark analysis was conducted to perform the analyses.

When the analyses were repeated using a 5‐year landmark approach, the associations remained consistent across all dementia outcomes and dietary indices (Figure 3 and Table S9).

## 4 DISCUSSION

Our study suggests that higher adherence to healthy dietary patterns is associated with a lower risk of dementia. However, the extent of these benefits varies according to the specific diet followed. Of the four dietary indices we assessed, the Mediterranean Diet exhibited the most consistent and robust associations across all dementia outcomes. In contrast, the MIND diet, despite being explicitly designed for dementia prevention,^24^ showed slightly weaker associations with all‐cause and vascular dementia and no significant link to Alzheimer disease risk. Interestingly, the Recommended Food Score (RFS) demonstrated associations similar in strength to those of the Mediterranean Diet. These findings have public health implications, as the Mediterranean Diet may not be feasible or culturally appropriate for everyone. Thus, offering diverse dietary patterns that emphasize various foods and nutrients could make dementia prevention strategies more accessible to global populations.

While prior research has established links between the Mediterranean and MIND diets with numerous health outcomes,^25^, ^26^ the association with dementia has been less thoroughly investigated for other dietary indices.^27^ Our study addresses this gap by exploring a broader range of dietary patterns. We observed weak to moderate correlations among the four dietary indices, reflecting both shared and unique features of healthy eating. Although some overlap exists, each diet emphasizes different aspects. The RFS diet emphasizes a wide variety of nutrient‐dense foods, including fruits, vegetables, whole grains, lean proteins and low‐fat dairy, without being tied to a specific cultural dietary pattern. Meanwhile, the MIND diet is a hybrid of the Mediterranean and DASH diets, designed specifically to promote brain health by emphasizing the intake of leafy greens, berries, nuts, and limiting foods associated with neurodegeneration, like red meat and sweets. This flexibility in the RFS and MIND diets may offer a more adaptable approach for populations where the Mediterranean Diet is less practical.

We found that adherence to RFS, MDS and, to a lesser extent, the MIND diet was associated with a lower risk of vascular dementia after adjusting for important dementia risk factors. This highlights the potential cerebrovascular benefits of specific dietary patterns, although research in this area remains limited.^5^ Diets rich in fruits, vegetables, whole grains, nuts and healthy fats may improve endothelial function, reduce oxidative stress and lower inflammation, thereby enhancing brain health and reducing dementia risk.^28^ In contrast, the HDI showed no association with dementia outcomes. This may be because the HDI is based on World Health Organization guidelines, which promote a balanced diet focusing on overall health—emphasizing fruits, vegetables, whole grains and legumes, while limiting saturated fats, added sugars and sodium—rather than specifically targeting neuroprotective foods. This finding is consistent with previous research showing no significant association between HDI adherence and reduced cognitive decline in older populations across Europe and the Americas.^29^

Our findings for non‐vascular dementia revealed consistent associations across several dietary indices, including the RFS, MIND diet and Mediterranean diet. The observed differences between the MIND and Mediterranean diets may be attributed to the broader nutritional profile of the Mediterranean diet, which typically includes more frequent consumption of fatty fish (rich in omega‐3 fatty acids), olive oil and a diverse range of fruits and vegetables.^30^ These components are abundant in antioxidants and anti‐inflammatory compounds, which may confer more robust neuroprotective effects compared with the MIND diet's more targeted food groups.^30^ These results align with previous research highlighting the cognitive benefits of Mediterranean and high‐fibre dietary patterns.^31^ Notably, higher adherence to both the RFS and MDS was associated with a reduced risk of Alzheimer disease, even after adjusting for sociodemographic and health‐related variables. This supports existing evidence of the Mediterranean diet's neuroprotective role in lowering Alzheimer disease incidence.^28^, ^32^ The beneficial effects of these diets are likely driven by their emphasis on neuroprotective and anti‐inflammatory nutrients, such as essential fatty acids and antioxidants, which support neuronal integrity and reduce systemic inflammation.^33^ Nevertheless, while our findings suggest a potential protective role of the RFS against dementia, the current evidence base remains limited and warrants further investigation.^34^

This study provides novel insights by simultaneously analysing multiple dietary patterns, addressing a research gap where dietary indices are often studied in isolation. Strengths include the study's prospective design, a large sample size, and an extended follow‐up period of nearly 10 years. However, there are limitations. First, although we minimized reverse causality by excluding dementia cases diagnosed within the first 5 years of follow‐up, dietary data were self‐reported and therefore susceptible to recall bias. Nevertheless, the prospective design reduces the risk of differential misclassification. Second, dementia diagnoses were ascertained through hospital inpatient records and linked data, which may underrepresent milder cases and lead to potential misclassification of dementia subtypes. Third, the UK Biobank cohort is not fully representative of the general UK population, potentially limiting the generalizability of our findings to other demographic or socioeconomic contexts. Fourth, we used complete case analysis and did not perform imputation for missing data. While this approach avoids the assumptions inherent in multiple imputation, it may have introduced selection bias and reduced statistical power. Fifth, given the low incidence of dementia cases observed in the cohort, there is a potential for small sample bias. Sixth, as this is an observational study, we cannot infer causality. Residual confounding and reverse causation remain possible, and future studies, such as Mendelian randomization, are needed to prove causality. Although several randomized controlled trials have been conducted to evaluate the impact of the Mediterranean diet or MIND diet on dementia‐related outcomes and cognitive function, the current evidence remains limited and inconclusive, with mixed findings across studies.^35^, ^36^, ^37^ Finally, the inclusion of a large number of covariates with multiple categories in the fully adjusted model may have introduced overfitting or reduced degrees of freedom, and although we adjusted for several sociodemographic, lifestyle and health‐related confounders, unmeasured variables such as genetic predisposition and baseline cognitive performance were not included and may have influenced the observed associations. While we selected covariates based on theoretical relevance and prior literature, and conducted sensitivity analyses with more parsimonious models (yielding consistent results), this remains a limitation that should be considered when interpreting the findings.

## 5 CONCLUSION

Our study provides evidence that adherence to healthy dietary patterns, particularly the Mediterranean Diet, MIND, and the Recommended Food Score, is associated with a lower risk of dementia, including vascular and non‐vascular types. These findings underscore the potential neuroprotective and cerebrovascular benefits of diets rich in fruits, vegetables, whole grains, nuts and healthy fats. Therefore, the Mediterranean and similar diets could be considered as promising models for future research to better understand whether and how specific biological mechanisms are activated that may contribute to improved brain health in older adults. This research highlights the importance of dietary quality and diversity in dementia prevention and suggests that adopting flexible, culturally adaptable dietary patterns may help make prevention strategies more accessible on a global scale. Future studies should focus on further elucidating the mechanisms behind these associations, and evidence addressing causality is needed as well as assessing the impact of diet on dementia risk in more diverse populations, given the limitations related to self‐reported dietary data and the demographic characteristics of the UK Biobank cohort.

## FUNDING INFORMATION

No financial disclosures were reported by the authors of this paper.

## CONFLICT OF INTEREST STATEMENT

The authors declare no conflicts of interest.

## PEER REVIEW

The peer review history for this article is available at https://www.webofscience.com/api/gateway/wos/peer‐review/10.1111/dom.16609.

## CONSENT

This study was conducted according to the guidelines laid down in the Declaration of Helsinki and all procedures involving research study participants were approved by the Northwest Multi‐Centre Research Ethics Committee (NHS National Research Ethics Service 16/NW/0274). Written informed consent was obtained from all subjects/patients.

## Supporting information


**Data S1.** Supporting Information.

## Data Availability

The data that support the findings of this study are available from UK Biobank. Restrictions apply to the availability of these data, which were used under license for this study. Data are available from UK Biobank (https://www.ukbiobank.ac.uk) with the permission of UK Biobank.

## References

[1] Livingston G , Sommerlad A , Orgeta V , et al. Dementia prevention, intervention, and care. Lancet. 2017;390(10113):2673‐2734. 28735855 10.1016/S0140-6736(17)31363-6

[2] Feigin VL , Abajobir AA , Abate KH , et al. Global, regional, and national burden of neurological disorders during 1990–2015: a systematic analysis for the Global Burden of Disease Study 2015. Lancet Neurol. 2017;16(11):877‐897. 28931491 10.1016/S1474-4422(17)30299-5 PMC5641502

[3] Livingston G , Huntley J , Sommerlad A , et al. Dementia prevention, intervention, and care: 2020 report of the Lancet Commission. Lancet. 2020;396(10248):413‐446. 32738937 10.1016/S0140-6736(20)30367-6 PMC7392084

[4] Wallace L , Brayne C . The need for a better global dementia response. Lancet Neurol. 2022;21(2):115‐116. 10.1016/S1474-4422(21)00459-2 34922642

[5] Livingston G , Huntley J , Liu KY , et al. Dementia prevention, intervention, and care: 2024 report of theLancet standing Commission. Lancet. 2024;404(10452):572‐628. 39096926 10.1016/S0140-6736(24)01296-0

[6] Hersi M , Irvine B , Gupta P , Gomes J , Birkett N , Krewski D . Risk factors associated with the onset and progression of Alzheimer's disease: a systematic review of the evidence. Neurotoxicology. 2017;61:143‐187. 28363508 10.1016/j.neuro.2017.03.006

[7] Tapsell LC , Neale EP , Satija A , Hu FB . Foods, nutrients, and dietary patterns: interconnections and implications for dietary guidelines. Adv Nutr. 2016;7(3):445‐454. 27184272 10.3945/an.115.011718 PMC4863273

[8] Zhang J , Cao X , Li X , et al. Associations of midlife dietary patterns with incident dementia and brain structure: findings from the UK biobank study. Am J Clin Nutr. 2023;118(1):218‐227. 37150507 10.1016/j.ajcnut.2023.05.007

[9] Livingstone KM , Abbott G , Bowe SJ , Ward J , Milte C , McNaughton SA . Diet quality indices, genetic risk and risk of cardiovascular disease and mortality: a longitudinal analysis of 77 004 UK biobank participants. BMJ Open. 2021;11(4):e045362. 10.1136/bmjopen-2020-045362 PMC8023730 33795309

[10] Jankovic N , Geelen A , Streppel MT , et al. Adherence to a healthy diet according to the World Health Organization guidelines and all‐cause mortality in elderly adults from Europe and the United States. Am J Epidemiol. 2014;180(10):978‐988. 25318818 10.1093/aje/kwu229 PMC4224363

[11] Ortolá R , Sotos‐Prieto M , García‐Esquinas E , Galán I , Rodríguez‐Artalejo F . Alcohol consumption patterns and mortality among older adults with health‐related or socioeconomic risk factors. JAMA Netw Open. 2024;7(8):e2424495‐e. 39133491 10.1001/jamanetworkopen.2024.24495 PMC11320169

[12] Morris MC , Tangney CC , Wang Y , Barnes LL , Bennett D , Aggarwal N . 02‐02‐04: mind diet score more predictive than dash or mediterranean diet scores. Alzheimers Dement. 2014;10(4S_Part_2):P166.

[13] Shi Y , Lin F , Li Y , et al. Association of pro‐inflammatory diet with increased risk of all‐cause dementia and Alzheimer's dementia: a prospective study of 166,377 UK biobank participants. BMC Med. 2023;21(1):266. 37480061 10.1186/s12916-023-02940-5 PMC10362711

[14] Sabia S , Dugravot A , Dartigues J‐F , et al. Physical activity, cognitive decline, and risk of dementia: 28 year follow‐up of Whitehall II cohort study. BMJ. 2017;357:j2709. 28642251 10.1136/bmj.j2709 PMC5480222

[15] Martínez‐González MA , Fernández‐Jarne E , Serrano‐Martínez M , Wright M , Gomez‐Gracia E . Development of a short dietary intake questionnaire for the quantitative estimation of adherence to a cardioprotective Mediterranean diet. Eur J Clin Nutr. 2004;58(11):1550‐1552. 15162136 10.1038/sj.ejcn.1602004

[16] Schröder H , Fitó M , Estruch R , et al. A short screener is valid for assessing Mediterranean diet adherence among older Spanish men and women. J Nutr. 2011;141(6):1140‐1145. 21508208 10.3945/jn.110.135566

[17] Bradbury KE , Young HJ , Guo W , Key TJ . Dietary assessment in UK biobank: an evaluation of the performance of the touchscreen dietary questionnaire. J Nutr Sci. 2018;7:e6. 29430297 10.1017/jns.2017.66 PMC5799609

[18] Maynard M , Gunnell D , Ness AR , Abraham L , Bates CJ , Blane D . What influences diet in early old age? Prospective and cross‐sectional analyses of the Boyd Orr cohort. Eur J Public Health. 2005;16(3):315‐323. 10.1093/eurpub/cki167 16141299

[19] Morris MC , Tangney CC , Wang Y , Sacks FM , Bennett DA , Aggarwal NT . MIND diet associated with reduced incidence of Alzheimer's disease. Alzheimers Dement. 2015;11(9):1007‐1014. 25681666 10.1016/j.jalz.2014.11.009 PMC4532650

[20] Statistics UIf . International Standard Classification of Education (ISCED) 2011 . 2012.

[21] WHO . Global Status Report on Alcohol and Health 2018 . 2018.

[22] Craig CL , Marshall AL , Sjöström M , et al. International physical activity questionnaire: 12‐country reliability and validity. Med Sci Sports Exerc. 2003;35(8):1381‐1395. 12900694 10.1249/01.MSS.0000078924.61453.FB

[23] Obesity WHOCo , World Health Organization . Obesity: Preventing and Managing the Global Epidemic: Report of a WHO Consultation. World Health Organization; 2000. 11234459

[24] Morris MC , Tangney CC , Wang Y , et al. MIND diet slows cognitive decline with aging. Alzheimers Dement. 2015;11(9):1015‐1022. 26086182 10.1016/j.jalz.2015.04.011 PMC4581900

[25] Sofi F , Macchi C , Abbate R , Gensini GF , Casini A . Mediterranean diet and health status: an updated meta‐analysis and a proposal for a literature‐based adherence score. Public Health Nutr. 2014;17(12):2769‐2782. 24476641 10.1017/S1368980013003169 PMC10282340

[26] Kheirouri S , Alizadeh M . MIND diet and cognitive performance in older adults: a systematic review. Crit Rev Food Sci Nutr. 2022;62(29):8059‐8077. 33989093 10.1080/10408398.2021.1925220

[27] Hu FB . Dietary pattern analysis: a new direction in nutritional epidemiology. Curr Opin Lipidol. 2002;13(1):3‐9. 11790957 10.1097/00041433-200202000-00002

[28] Barnard ND , Bush AI , Ceccarelli A , et al. Dietary and lifestyle guidelines for the prevention of Alzheimer's disease. Neurobiol Aging. 2014;35:S74‐S78. 24913896 10.1016/j.neurobiolaging.2014.03.033

[29] Berendsen AA , Kang JH , van de Rest O , et al. Association of Adherence to a healthy diet with cognitive decline in European and American older adults: a meta‐analysis within the CHANCES consortium. Dement Geriatr Cogn Disord. 2017;43(3–4):215‐227. 28324877 10.1159/000464269 PMC5447176

[30] Agarwal P , Leurgans SE , Agrawal S , et al. Association of Mediterranean‐DASH intervention for neurodegenerative delay and Mediterranean diets with Alzheimer disease pathology. Neurology. 2023;100(22):e2259‐e2268. 36889921 10.1212/WNL.0000000000207176 PMC10259273

[31] Sofi F , Cesari F , Abbate R , Gensini GF , Casini A . Adherence to Mediterranean diet and health status: meta‐analysis. BMJ. 2008;337:a1344. 18786971 10.1136/bmj.a1344 PMC2533524

[32] Scheltens P , Blennow K , Breteler MM , et al. Alzheimer's disease. Lancet. 2016;388(10043):505‐517. 26921134 10.1016/S0140-6736(15)01124-1

[33] Yassine HN , Samieri C , Livingston G , et al. Nutrition state of science and dementia prevention: recommendations of the nutrition for dementia prevention working group. Lancet Healthy Longev. 2022;3(7):e501‐e512. 35821792 10.1016/s2666-7568(22)00120-9 PMC9273104

[34] van de Rest O , Berendsen AAM , Haveman‐Nies A , de Groot LC . Dietary patterns, cognitive decline, and dementia: a systematic review. Adv Nutr. 2015;6(2):154‐168. 25770254 10.3945/an.114.007617 PMC4352174

[35] Barnes LL , Dhana K , Liu X , et al. Trial of the MIND diet for prevention of cognitive decline in older persons. N Engl J Med. 2023;389(7):602‐611. 37466280 10.1056/NEJMoa2302368 PMC10513737

[36] Valls‐Pedret C , Sala‐Vila A , Serra‐Mir M , et al. Mediterranean diet and age‐related cognitive decline: a randomized clinical trial. JAMA Intern Med. 2015;175(7):1094‐1103. 25961184 10.1001/jamainternmed.2015.1668

[37] Fu J , Tan L‐J , Lee JE , Shin S . Association between the mediterranean diet and cognitive health among healthy adults: a systematic review and meta‐analysis. Front Nutr. 2022;9:2022. 10.3389/fnut.2022.946361 PMC9372716 35967772

