# Supplementary material for: Adherence to four dietary indices and the risk of all‐cause and cause‐specific dementia: Findings from the UK Biobank study
Source: Diabetes Obes Metab. 2025 Jul 16;27(10):5612–21. doi: 10.1111/dom.16609 (PMC12409230; doi:10.1111/dom.16609)
Supplement: Supplementary file 1 — Data S1. Supporting Information. [file DOM-27-5612-s001.docx]

**SUPPLEMENTARY MATERIAL**

**Figure S1.** Flow chart of participants included in the study.

UK Biobank participants

*n* = 502,520

- n = 502,535

Excluded (*n* = 380,999)

Participants with missing data for any dietary index.502,535

Missing covariate data (*n* = 1848)

- n = 502,535

Data available for analysis

*n* = 123,369

- n = 502,535

Data available for analysis

*n* = 121,521

- n = 502,535

Table S1. Cut off points for quartiles of adherence to each of the dietary indices.

|  | Mean | Q1 | Q2 | Q3 | Q4 | Q5 |
| --- | --- | --- | --- | --- | --- | --- |
| **MDS** | 2.30 (1.10) | 1 | 2 | 3 | 4-5 | 6 -9 |
| **HDI** | 2.78 (1.44) | 1-2 | 3 | 4-5 | 6 | 7-10 |
| **RFS** | 2.74 (1.39) | 1-4 | 5-6 | 7-8 | 9-11 | 12-21 |
| **MIND** | 2.61 (1.48) | 2-5 | 5,5 | 6 | 6.5-7 | 7.5-10.5 |

Table S2. Cohort characteristics by levels of adherence to MIND diet.

|  | Lowest adherence | Moderately low adherence | Moderately high adherence | Highest adherence |
| --- | --- | --- | --- | --- |
| Total n | 33,511 (27.6%) | 42,230 (34.8%) | 19,070 (15.7%) | 26,710 (22.0%) |
| **Age** (years) | 55.24 (8.16) | 55.73 (8.02) | 56.01 (7.84) | 56.43 (7.75) |
| **Sex** |  |  |  |  |
| Women | 16,502 (49.2%) | 22,355 (52.9%) | 10,622 (55.7%) | 15,478 (57.9%) |
| Men | 17,009 (50.8%) | 19,875 (47.1%) | 8,448 (44.3%) | 11,232 (42.1%) |
| **Ethnicity** |  |  |  |  |
| White | 32,053 (95.6%) | 40,514 (95.9%) | 18,429 (96.6%) | 25,823 (96.7%) |
| Mixed | 397 (1.2%) | 566 (1.3%) | 245 (1.3%) | 321 (1.2%) |
| South Asian | 602 (1.8%) | 583 (1.4%) | 198 (1.0%) | 268 (1.0%) |
| Black | 378 (1.1%) | 459 (1.1%) | 160 (0.8%) | 222 (0.8%) |
| Chinese | 81 (0.2%) | 108 (0.3%) | 38 (0.2%) | 76 (0.3%) |
| **Education** |  |  |  |  |
| None of the above | 3,913 (11.7%) | 3,442 (8.2%) | 1,158 (6.1%) | 1,342 (5.0%) |
| CSEs | 2,133 (6.4%) | 1,843 (4.4%) | 605 (3.2%) | 742 (2.8%) |
| O-levels | 8,941 (26.7%) | 9,875 (23.4%) | 3,925 (20.6%) | 5,000 (18.7%) |
| A-levels | 4,900 (14.6%) | 6,421 (15.2%) | 2,805 (14.7%) | 3,843 (14.4%) |
| College/University degree | 13,624 (40.7%) | 20,649 (48.9%) | 10,577 (55.5%) | 15,783 (59.1%) |
| **Smoking** |  |  |  |  |
| Never | 19,248 (57.5%) | 23,805 (56.5%) | 10,712 (56.3%) | 14,910 (55.9%) |
| Previous | 11,263 (33.7%) | 15,167 (36.0%) | 7,089 (37.2%) | 10,190 (38.2%) |
| Current | 2,943 (8.8%) | 3,197 (7.6%) | 1,239 (6.5%) | 1,582 (5.9%) |
| **Alcohol consumption** (Units/week) | 14.87 (18.24) | 16.06 (17.12) | 17.20 (16.64) | 17.66 (16.49) |
| **Physical activity** |  |  |  |  |
| Inactive | 6,938 (20.7%) | 7,601 (18.0%) | 3,173 (16.6%) | 4,103 (15.4%) |
| Moderate active | 13,943 (41.6%) | 18,141 (43.0%) | 8,223 (43.1%) | 11,525 (43.1%) |
| Active | 12,630 (37.7%) | 16,488 (39.0%) | 7,674 (40.2%) | 11,082 (41.5%) |
| **Family/friends visits** |  |  |  |  |
| Almost daily | 3,360 (10.0%) | 3,881 (9.2%) | 1,650 (8.7%) | 2,362 (8.8%) |
| 2-4 times a week | 9,738 (29.1%) | 12,396 (29.4%) | 5,614 (29.4%) | 8,020 (30.0%) |
| About once a week | 12,190 (36.4%) | 15,875 (37.6%) | 7,232 (37.9%) | 10,076 (37.7%) |
| About once a month | 5,107 (15.2%) | 6,536 (15.5%) | 3,080 (16.2%) | 4,247 (15.9%) |
| Once every few months | 2,550 (7.6%) | 2,990 (7.1%) | 1,302 (6.8%) | 1,746 (6.5%) |
| Never or almost never | 506 (1.5%) | 498 (1.2%) | 177 (0.9%) | 227 (0.8%) |
| No friends/family | 60 (0.2%) | 54 (0.1%) | 15 (0.1%) | 32 (0.1%) |
| **Leisure social activities** |  |  |  |  |
| None of the above | 10,027 (29.9%) | 11,813 (28.0%) | 5,114 (26.8%) | 7,041 (26.4%) |
| Sports club or gym | 10,357 (30.9%) | 14,453 (34.2%) | 7,060 (37.0%) | 10,463 (39.2%) |
| Pub or social club | 6,056 (18.1%) | 6,644 (15.7%) | 2,787 (14.6%) | 3,524 (13.2%) |
| Religious group | 3,299 (9.8%) | 4,010 (9.5%) | 1,614 (8.5%) | 2,143 (8.0%) |
| Adult education class | 846 (2.5%) | 1,388 (3.3%) | 678 (3.6%) | 1,059 (4.0%) |
| Other group activity | 2,926 (8.7%) | 3,922 (9.3%) | 1,817 (9.5%) | 2,480 (9.3%) |
| **Air pollution, PM 2.5** (micro-g/m^3^) | 9.93 (1.01) | 9.90 (1.02) | 9.90 (1.05) | 9.90 (1.06) |
| **Body mass index** (kg.m^2^) | 27.40 (4.80) | 26.77 (4.47) | 26.31 (4.31) | 26.08 (4.21) |
| **CVD medication** |  |  |  |  |
| No | 28,057 (83.7%) | 35,511 (84.1%) | 16,144 (84.7%) | 22,708 (85.0%) |
| Yes | 5,454 (16.3%) | 6,719 (15.9%) | 2,926 (15.3%) | 4,002 (15.0%) |
| **Type 2 Diabetes** |  |  |  |  |
| No | 31,951 (95.3%) | 40,608 (96.2%) | 18,441 (96.7%) | 25,867 (96.8%) |
| Yes | 1,560 (4.7%) | 1,622 (3.8%) | 629 (3.3%) | 843 (3.2%) |
| **Hypertension** |  |  |  |  |
| No | 25,508 (76.1%) | 32,558 (77.1%) | 14,889 (78.1%) | 21,037 (78.8%) |
| Yes | 8,003 (23.9%) | 9,672 (22.9%) | 4,181 (21.9%) | 5,673 (21.2%) |
| **Depression** |  |  |  |  |
| No | 31,602 (94.3%) | 40,158 (95.1%) | 18,205 (95.5%) | 25,621 (95.9%) |
| Yes | 1,909 (5.7%) | 2,072 (4.9%) | 865 (4.5%) | 1,089 (4.1%) |
| **LDL cholesterol** (mmol/L) | 3.53 (0.85) | 3.55 (0.85) | 3.55 (0.84) | 3.57 (0.84) |
| **Traumatic Brain Injury** |  |  |  |  |
| No | 33,339 (99.5%) | 42,042 (99.6%) | 18,988 (99.6%) | 26,587 (99.5%) |
| Yes | 172 (0.5%) | 188 (0.4%) | 82 (0.4%) | 123 (0.5%) |
| **Hearing problems** |  |  |  |  |
| No | 24,905 (74.3%) | 31,516 (74.6%) | 14,385 (75.4%) | 20,035 (75.0%) |
| Yes | 8,593 (25.6%) | 10,710 (25.4%) | 4,682 (24.6%) | 6,670 (25.0%) |

Data presented as mean and standard deviation for continuous variables and as frequency and % for categorical variables. MDS: Mediterranean Diet Score; BMI: body mass index; IPAQ: International Physical Activity Questionnaire.

Table S3. Cohort characteristics by levels of adherence to RFS diet.

|  | Lowest adherence | Moderately low adherence | Moderately high adherence | Highest adherence |
| --- | --- | --- | --- | --- |
| Total n | 35,375 (29.1%) | 33,267 (27.4%) | 27,999 (23.0%) | 24,880 (20.5%) |
| **Age** (years) | 54.74 (8.23) | 55.52 (8.07) | 56.19 (7.82) | 57.20 (7.44) |
| **Sex** |  |  |  |  |
| Women | 17,330 (49.0%) | 17,047 (51.2%) | 15,219 (54.4%) | 15,361 (61.7%) |
| Men | 18,045 (51.0%) | 16,220 (48.8%) | 12,780 (45.6%) | 9,519 (38.3%) |
| **Ethnicity** |  |  |  |  |
| White | 33,506 (94.7%) | 31,937 (96.0%) | 27,139 (96.9%) | 24,237 (97.4%) |
| Mixed | 523 (1.5%) | 400 (1.2%) | 322 (1.2%) | 284 (1.1%) |
| South Asian | 734 (2.1%) | 482 (1.4%) | 260 (0.9%) | 175 (0.7%) |
| Black | 517 (1.5%) | 355 (1.1%) | 221 (0.8%) | 126 (0.5%) |
| Chinese | 95 (0.3%) | 93 (0.3%) | 57 (0.2%) | 58 (0.2%) |
| **Education** |  |  |  |  |
| None of the above | 3,932 (11.1%) | 2,729 (8.2%) | 1,834 (6.6%) | 1,360 (5.5%) |
| CSEs | 2,324 (6.6%) | 1,448 (4.4%) | 952 (3.4%) | 599 (2.4%) |
| O-levels | 9,153 (25.9%) | 7,820 (23.5%) | 5,951 (21.3%) | 4,817 (19.4%) |
| A-levels | 5,179 (14.6%) | 4,873 (14.6%) | 4,193 (15.0%) | 3,724 (15.0%) |
| College/University degree | 14,787 (41.8%) | 16,397 (49.3%) | 15,069 (53.8%) | 14,380 (57.8%) |
| **Smoking** |  |  |  |  |
| Never | 19,226 (54.4%) | 18,618 (56.0%) | 16,103 (57.6%) | 14,728 (59.3%) |
| Previous | 12,528 (35.5%) | 11,975 (36.0%) | 10,173 (36.4%) | 9,033 (36.3%) |
| Current | 3,560 (10.1%) | 2,632 (7.9%) | 1,678 (6.0%) | 1,091 (4.4%) |
| **Alcohol consumption** (Units/week) | 17.79 (19.33) | 16.81 (17.65) | 15.80 (16.16) | 13.89 (14.28) |
| **Physical activity** |  |  |  |  |
| Inactive | 7,274 (20.6%) | 6,200 (18.6%) | 4,862 (17.4%) | 3,479 (14.0%) |
| Moderate active | 14,890 (42.1%) | 14,106 (42.4%) | 12,215 (43.6%) | 10,621 (42.7%) |
| Active | 13,211 (37.3%) | 12,961 (39.0%) | 10,922 (39.0%) | 10,780 (43.3%) |
| **Family/friends visits** |  |  |  |  |
| Almost daily | 3,337 (9.4%) | 3,074 (9.2%) | 2,482 (8.9%) | 2,360 (9.5%) |
| 2-4 times a week | 10,170 (28.7%) | 9,738 (29.3%) | 8,231 (29.4%) | 7,629 (30.7%) |
| About once a week | 13,402 (37.9%) | 12,389 (37.2%) | 10,500 (37.5%) | 9,082 (36.5%) |
| About once a month | 5,377 (15.2%) | 5,198 (15.6%) | 4,461 (15.9%) | 3,934 (15.8%) |
| Once every few months | 2,531 (7.2%) | 2,454 (7.4%) | 1,981 (7.1%) | 1,622 (6.5%) |
| Never or almost never | 504 (1.4%) | 372 (1.1%) | 305 (1.1%) | 227 (0.9%) |
| No friends/family | 54 (0.2%) | 42 (0.1%) | 39 (0.1%) | 26 (0.1%) |
| **Leisure social activities** |  |  |  |  |
| None of the above | 10,565 (29.9%) | 9,390 (28.2%) | 7,701 (27.5%) | 6,339 (25.5%) |
| Sports club or gym | 11,294 (31.9%) | 11,508 (34.6%) | 10,025 (35.8%) | 9,506 (38.2%) |
| Pub or social club | 6,688 (18.9%) | 5,433 (16.3%) | 3,957 (14.1%) | 2,933 (11.8%) |
| Religious group | 2,901 (8.2%) | 3,002 (9.0%) | 2,684 (9.6%) | 2,479 (10.0%) |
| Adult education class | 927 (2.6%) | 1,019 (3.1%) | 960 (3.4%) | 1,065 (4.3%) |
| Other group activity | 3,000 (8.5%) | 2,915 (8.8%) | 2,672 (9.5%) | 2,558 (10.3%) |
| **Air pollution, PM 2.5** (micro-g/m^3^) | 9.93 (1.02) | 9.91 (1.04) | 9.90 (1.05) | 9.87 (1.01) |
| **Body mass index** (kg.m^2^) | 27.14 (4.57) | 26.86 (4.54) | 26.52 (4.44) | 26.15 (4.40) |
| **CVD medication** |  |  |  |  |
| No | 29,787 (84.2%) | 28,047 (84.3%) | 23,560 (84.1%) | 21,026 (84.5%) |
| Yes | 5,588 (15.8%) | 5,220 (15.7%) | 4,439 (15.9%) | 3,854 (15.5%) |
| **Type 2 Diabetes** |  |  |  |  |
| No | 33,948 (96.0%) | 31,861 (95.8%) | 26,979 (96.4%) | 24,079 (96.8%) |
| Yes | 1,427 (4.0%) | 1,406 (4.2%) | 1,020 (3.6%) | 801 (3.2%) |
| **Hypertension** |  |  |  |  |
| No | 27,170 (76.8%) | 25,793 (77.5%) | 21,660 (77.4%) | 19,369 (77.8%) |
| Yes | 8,205 (23.2%) | 7,474 (22.5%) | 6,339 (22.6%) | 5,511 (22.2%) |
| **Depression** |  |  |  |  |
| No | 33,436 (94.5%) | 31,633 (95.1%) | 26,732 (95.5%) | 23,785 (95.6%) |
| Yes | 1,939 (5.5%) | 1,634 (4.9%) | 1,267 (4.5%) | 1,095 (4.4%) |
| **LDL cholesterol** (mmol/L) | 3.56 (0.85) | 3.55 (0.85) | 3.54 (0.84) | 3.55 (0.84) |
| **Traumatic Brain Injury** |  |  |  |  |
| No | 35,185 (99.5%) | 33,125 (99.6%) | 27,881 (99.6%) | 24,765 (99.5%) |
| Yes | 190 (0.5%) | 142 (0.4%) | 118 (0.4%) | 115 (0.5%) |
| **Hearing problems** |  |  |  |  |
| No | 26,387 (74.6%) | 24,832 (74.6%) | 20,917 (74.7%) | 18,705 (75.2%) |
| Yes | 8,978 (25.4%) | 8,430 (25.3%) | 7,078 (25.3%) | 6,169 (24.8%) |

Data presented as mean and standard deviation for continuous variables and as frequency and % for categorical variables. MDS: Mediterranean Diet Score; BMI: body mass index; IPAQ: International Physical Activity Questionnaire.

Table S4. Cohort characteristics levels of adherence to HDI diet.

|  | Lowest adherence | Moderately low adherence | Moderately high adherence | Highest adherence |
| --- | --- | --- | --- | --- |
| Total n | 48,725 (40.1%) | 31,973 (26.3%) | 23,981 (19.7%) | 16,842 (13.9%) |
| **Age** (years) | 55.49 (7.98) | 55.77 (8.04) | 56.13 (8.00) | 56.24 (7.82) |
| **Sex** |  |  |  |  |
| Women | 26,888 (55.2%) | 16,624 (52.0%) | 12,280 (51.2%) | 9,165 (54.4%) |
| Men | 21,837 (44.8%) | 15,349 (48.0%) | 11,701 (48.8%) | 7,677 (45.6%) |
| **Ethnicity** |  |  |  |  |
| White | 46,804 (96.1%) | 30,815 (96.4%) | 23,098 (96.3%) | 16,102 (95.6%) |
| Mixed | 612 (1.3%) | 378 (1.2%) | 301 (1.3%) | 238 (1.4%) |
| South Asian | 608 (1.2%) | 399 (1.2%) | 340 (1.4%) | 304 (1.8%) |
| Black | 559 (1.1%) | 317 (1.0%) | 191 (0.8%) | 152 (0.9%) |
| Chinese | 142 (0.3%) | 64 (0.2%) | 51 (0.2%) | 46 (0.3%) |
| **Education** |  |  |  |  |
| None of the above | 4,129 (8.5%) | 2,704 (8.5%) | 1,887 (7.9%) | 1,135 (6.7%) |
| CSEs | 2,428 (5.0%) | 1,393 (4.4%) | 979 (4.1%) | 523 (3.1%) |
| O-levels | 11,693 (24.0%) | 7,414 (23.2%) | 5,230 (21.8%) | 3,404 (20.2%) |
| A-levels | 7,435 (15.3%) | 4,627 (14.5%) | 3,508 (14.6%) | 2,399 (14.2%) |
| College/University degree | 23,040 (47.3%) | 15,835 (49.5%) | 12,377 (51.6%) | 9,381 (55.7%) |
| **Smoking** |  |  |  |  |
| Never | 26,531 (54.5%) | 18,117 (56.7%) | 14,062 (58.7%) | 9,965 (59.3%) |
| Previous | 17,755 (36.5%) | 11,425 (35.8%) | 8,486 (35.4%) | 6,043 (35.9%) |
| Current | 4,360 (9.0%) | 2,393 (7.5%) | 1,403 (5.9%) | 805 (4.8%) |
| **Alcohol consumption** (Units/week) | 17.91 (18.28) | 16.27 (17.38) | 14.72 (16.16) | 13.68 (14.86) |
| **Physical activity** |  |  |  |  |
| Inactive | 10,070 (20.7%) | 5,763 (18.0%) | 3,766 (15.7%) | 2,216 (13.2%) |
| Moderate active | 21,106 (43.3%) | 13,719 (42.9%) | 10,113 (42.2%) | 6,894 (40.9%) |
| Active | 17,549 (36.0%) | 12,491 (39.1%) | 10,102 (42.1%) | 7,732 (45.9%) |
| **Family/friends visits** |  |  |  |  |
| Almost daily | 4,599 (9.4%) | 2,984 (9.3%) | 2,118 (8.8%) | 1,552 (9.2%) |
| 2-4 times a week | 14,195 (29.1%) | 9,502 (29.7%) | 7,132 (29.7%) | 4,939 (29.3%) |
| About once a week | 18,225 (37.4%) | 11,813 (36.9%) | 9,093 (37.9%) | 6,242 (37.1%) |
| About once a month | 7,588 (15.6%) | 5,019 (15.7%) | 3,708 (15.5%) | 2,655 (15.8%) |
| Once every few months | 3,472 (7.1%) | 2,269 (7.1%) | 1,621 (6.8%) | 1,226 (7.3%) |
| Never or almost never | 576 (1.2%) | 343 (1.1%) | 279 (1.2%) | 210 (1.2%) |
| No friends/family | 70 (0.1%) | 43 (0.1%) | 30 (0.1%) | 18 (0.1%) |
| **Leisure social activities** |  |  |  |  |
| None of the above | 14,223 (29.2%) | 8,963 (28.0%) | 6,341 (26.4%) | 4,468 (26.5%) |
| Sports club or gym | 16,278 (33.4%) | 11,079 (34.7%) | 8,644 (36.0%) | 6,332 (37.6%) |
| Pub or social club | 8,443 (17.3%) | 5,011 (15.7%) | 3,419 (14.3%) | 2,138 (12.7%) |
| Religious group | 4,018 (8.2%) | 3,021 (9.4%) | 2,437 (10.2%) | 1,590 (9.4%) |
| Adult education class | 1,506 (3.1%) | 989 (3.1%) | 819 (3.4%) | 657 (3.9%) |
| Other group activity | 4,257 (8.7%) | 2,910 (9.1%) | 2,321 (9.7%) | 1,657 (9.8%) |
| **Air pollution, PM 2.5** (micro-g/m^3^) | 9.91 (1.04) | 9.90 (1.03) | 9.89 (1.01) | 9.93 (1.02) |
| **Body mass index** (kg.m^2^) | 27.09 (4.61) | 26.74 (4.44) | 26.47 (4.46) | 25.94 (4.30) |
| **CVD medication** |  |  |  |  |
| No | 41,142 (84.4%) | 26,949 (84.3%) | 20,106 (83.8%) | 14,223 (84.4%) |
| Yes | 7,583 (15.6%) | 5,024 (15.7%) | 3,875 (16.2%) | 2,619 (15.6%) |
| **Type 2 Diabetes** |  |  |  |  |
| No | 46,779 (96.0%) | 30,727 (96.1%) | 23,060 (96.2%) | 16,301 (96.8%) |
| Yes | 1,946 (4.0%) | 1,246 (3.9%) | 921 (3.8%) | 541 (3.2%) |
| **Hypertension** |  |  |  |  |
| No | 37,427 (76.8%) | 24,717 (77.3%) | 18,619 (77.6%) | 13,229 (78.5%) |
| Yes | 11,298 (23.2%) | 7,256 (22.7%) | 5,362 (22.4%) | 3,613 (21.5%) |
| **Depression** |  |  |  |  |
| No | 46,415 (95.3%) | 30,345 (94.9%) | 22,775 (95.0%) | 16,051 (95.3%) |
| Yes | 2,310 (4.7%) | 1,628 (5.1%) | 1,206 (5.0%) | 791 (4.7%) |
| **LDL cholesterol** (mmol/L) | 3.59 (0.85) | 3.55 (0.85) | 3.52 (0.83) | 3.49 (0.83) |
| **Traumatic Brain Injury** |  |  |  |  |
| No | 48,501 (99.5%) | 31,817 (99.5%) | 23,875 (99.6%) | 16,763 (99.5%) |
| Yes | 224 (0.5%) | 156 (0.5%) | 106 (0.4%) | 79 (0.5%) |
| **Hearing problems** |  |  |  |  |
| No | 36,569 (75.1%) | 23,832 (74.5%) | 17,847 (74.4%) | 12,593 (74.8%) |
| Yes | 12,144 (24.9%) | 8,138 (25.5%) | 6,130 (25.6%) | 4,243 (25.2%) |

Data presented as mean and standard deviation for continuous variables and as frequency and % for categorical variables. MDS: Mediterranean Diet Score; BMI: body mass index; IPAQ: International Physical Activity Questionnaire.

**Table S5.** Associations of levels of adherence to Dietary Indices with All-cause dementia incidence.

|  |  | **Model 0** |  | **Model 1** |  | **Model 2** |  | **Model 3** |  |
| --- | --- | --- | --- | --- | --- | --- | --- | --- | --- |
| **MIND** | **Total n / cases** | **HR (95% CI)** | **p-value** | **HR (95% CI)** | **p-value** | **HR (95% CI)** | **p-value** | **HR (95% CI)** | **p-value** |
| Lowest adherence | 33,511 / 202 | 1.00 (ref.) |  | 1.00 (ref.) |  | 1.00 (ref.) |  | 1.00 (ref.) |  |
| Moderately low adherence | 42,230 / 228 | 0.88 (0.73; 1.06) | 0.186 | 0.86 (0.71; 1.03) | 0.119 | 0.88 (0.73; 1.06) | 0.181 | 0.88 (0.73; 1.06) | 0.203 |
| Moderately high adherence | 19,070 / 87 | 0.73 (0.57; 0.94) | 0.016 | 0.73 (0.57; 0.94) | 0.016 | 0.76 (0.59; 0.97) | 0.033 | 0.76 (0.59; 0.98) | 0.036 |
| Highest adherence | 26,710 / 104 | 0.65 (0.52; 0.82) | <0.0001 | 0.63 (0.50; 0.80) | <0.0001 | 0.66 (0.53; 0.84) | 0.001 | 0.67 (0.53; 0.85) | 0.001 |
| Trend | 121,521 / 621 | 0.86 (0.80; 0.93) | <0.0001 | 0.86 (0.80;0.92) | <0.0001 | 0.87 (0.81; 0.94) | <0.0001 | 0.87 (0.81; 0.94) | <0.0001 |
| **MDS** |  |  |  |  |  |  |  |  |  |
| Lowest adherence | 6,556 / 35 | 1.00 (ref.) |  | 1.00 (ref.) |  | 1.00 (ref.) |  | 1.00 (ref.) |  |
| Moderately low adherence | 42,912 / 268 | 0.64 (0.56; 0.74) | <0.0001 | 0.76 (0.66; 0.87) | <0.0001 | 0.75 (0.63; 0.88) | 0.001 | 0.75 (0.64; 0.89) | 0.001 |
| Moderately high adherence | 27,697 / 125 | 0.46 (0.38; 0.55) | <0.0001 | 0.54 (0.44; 0.65) | <0.0001 | 0.53 (0.43; 0.65) | <0.0001 | 0.54 (0.44; 0.66) | <0.0001 |
| Highest adherence | 44,356 / 193 | 0.44 (0.37; 0.55) | <0.0001 | 0.52 (0.44; 0.62) | <0.0001 | 0.53 (0.44; 0.63) | <0.0001 | 0.53 (0.45; 0.63) | <0.0001 |
| Trend | 121,521 / 621 | 0.74 (0.70; 0.78) | <0.0001 | 0.79 (0.75; 0.83) | <0.0001 | 0.79 (0.75; 0.84) | <0.0001 | 0.79 (0.75; 0.84) | <0.0001 |
| **RFS** |  |  |  |  |  |  |  |  |  |
| Lowest adherence | 35,375 / 196 | 1.00 (ref.) |  | 1.00 (ref.) |  | 1.00 (ref.) |  | 1.00 (ref.) |  |
| Moderately low adherence | 33,267 / 179 | 0.91 (0.75; 1.11) | 0.369 | 0.85 (0.70; 1.04) | 0.128 | 0.86 (0.71; 1.05) | 0.152 | 0.86 (0.71; 1.05) | 0.151 |
| Moderately high adherence | 27,999 / 144 | 0.87 (0.70; 1.07) | 0.195 | 0.79 (0.64; 0.98) | 0.034 | 0.80 (0.64; 0.99) | 0.042 | 0.80 (0.65; 0.99) | 0.042 |
| Highest adherence | 24,880 / 102 | 0.70 (0.55; 0.89) | 0.004 | 0.61 (0.48; 0.77) | <0.0001 | 0.61 (0.48; 0.78) | <0.0001 | 0.61 (0.48; 0.78) | <0.0001 |
| Trend | 121,521 / 621 | 0.90 (0.84; 0.96) | 0.004 | 0.86 (0.80; 0.92) | <0.0001 | 0.86 (0.80; 0.92) | <0.0001 | 0.86 (0.80; 0.92) | <0.0001 |
| **HDI** |  |  |  |  |  |  |  |  |  |
| Lowest adherence | 48,725 / 237 | 1.00 (ref.) |  | 1.00 (ref.) |  | 1.00 (ref.) |  | 1.00 (ref.) |  |
| Moderately low adherence | 31,973 / 173 | 1.14 (0.94; 1.38) | 0.180 | 1.07 (0.88; 1.30) | 0.439 | 1.07 (0.88; 1.30) | 0.474 | 1.06 (0.88; 1.29) | 0.504 |
| Moderately high adherence | 23,981 / 131 | 1.13 (0.91; 1.40) | 0.241 | 1.03 (0.83; 1.27) | 0.777 | 1.02 (0.82; 1.26) | 0.834 | 1.00 (0.81; 1.24) | 0.931 |
| Highest adherence | 16,842 / 80 | 0.97 (0.76; 1.26) | 0.874 | 0.92 (0.71; 1.18) | 0.537 | 0.91 (0.71; 1.17) | 0.486 | 0.90 (0.70; 1.17) | 0.451 |
| Trend | 121,521 / 621 | 1.04 (0.94; 1.09) | 0.704 | 0.98 (0.91; 10.0) | 0.703 | 0.98 (0.91; 1.05) | 0.633 | 0.97 (0.90; 1.05) | 0.563 |

Data are presented as adjusted hazard ratios (HRs) with 95% confidence intervals (CIs). Low adherence to the dietary score (quartile 1) was used as the reference group. The trend represents the risk of dementia associated with each 1-quartile increase in adherence. Analyses were performed as follows: Model 0 was unadjusted; Model 1 was adjusted for sociodemographic factors (age, sex, education, and ethnicity); Model 2 was further adjusted for lifestyle-related factors (smoking, alcohol intake, physical activity, social interaction, and PM2.5 exposure); and Model 3 was additionally adjusted for health-related factors (BMI, hypertension, diabetes, depression, history of traumatic brain injury, hearing problems, LDL cholesterol, and cardiovascular medications).

**Table S6.** Associations of levels of adherence to Dietary Indices with Vascular dementia incidence.

|  |  | **Model 0** |  | **Model 1** |  | **Model 2** |  | **Model 3** |  |
| --- | --- | --- | --- | --- | --- | --- | --- | --- | --- |
| **MIND** | **Total n / cases** | **HR (95% CI)** | **p-value** | **HR (95% CI)** | **p-value** | **HR (95% CI)** | **p-value** | **HR (95% CI)** | **p-value** |
| Lowest adherence | 33,511 / 40 | 1.00 (ref.) |  | 1.00 (ref.) |  | 1.00 (ref.) |  | 1.00 (ref.) |  |
| Moderately low adherence | 42,230 / 50 | 0.95 (0.65; 1.41) | 0.813 | 0.98 (0.67; 1.45) | 0.935 | 1.01 (0.69; 1.50) | 0.947 | 1.04 (0.70; 1.54) | 0.843 |
| Moderately high adherence | 19,070 / 8 | 0.31 (0.15; 0.66) | 0.002 | 0.34 (0.16; 0.73) | 0.005 | 0.36 (0.17; 0.77) | 0.008 | 0.37 (0.18; 0.80) | 0.010 |
| Highest adherence | 26,710 / 20 | 0.56 (0.33; 0.95) | 0.031 | 0.62 (0.36; 0.95) | 0.040 | 0.66 (0.39; 0.96) | 0.042 | 0.68 (0.40; 0.97) | 0.045 |
| Trend | 121,521 / 118 | 0.77 (0.65; 0.91) | 0.003 | 0.80 (0.67; 0.95) | 0.010 | 0.82 (0.69; 0.97) | 0.021 | 0.83 (0.70; 0.98) | 0.030 |
| **MDS** |  |  |  |  |  |  |  |  |  |
| Lowest adherence | 6,556 / 12 | 1.00 (ref.) |  | 1.00 (ref.) |  | 1.00 (ref.) |  | 1.00 (ref.) |  |
| Moderately low adherence | 42,912 / 45 | 0.55 (0.41; 0.75) | <0.0001 | 0.71 (0.52; 0.97) | 0.029 | 0.68 (0.47; 0.97) | 0.034 | 0.69 (0.48; 0.99) | 0.044 |
| Moderately high adherence | 27,697 / 27 | 0.42 (0.28; 0.63) | <0.0001 | 0.54 (0.36; 0.82) | 0.004 | 0.53 (0.34; 0.81) | 0.004 | 0.54 (0.35; 0.83) | 0.005 |
| Highest adherence | 44,356 / 34 | 0.33 (023; 0.48) | <0.0001 | 0.45 (0.31; 0.66) | <0.0001 | 0.45 (0.30; 0.66) | <0.0001 | 0.46 (0.31; 0.68) | <0.0001 |
| Trend | 121,521 / 118 | 0.68 (0.60; 0.76) | <0.0001 | 0.76 (0.68; 0.85) | <0.0001 | 0.76 (0.67; 0.86) | <0.0001 | 0.77 (0.68; 0.87) | <0.0001 |
| **RFS** |  |  |  |  |  |  |  |  |  |
| Lowest adherence | 35,375 / 45 | 1.00 (ref.) |  | 1.00 (ref.) |  | 1.00 (ref.) |  | 1.00 (ref.) |  |
| Moderately low adherence | 33,267 / 24 | 0.56 (0.35; 0.90) | 0.015 | 0.54 (0.34; 0.87) | 0.010 | 0.55 (0.35; 0.88) | 0.012 | 0.55 (0.35; 0.88) | 0.012 |
| Moderately high adherence | 27,999 / 27 | 0.68 (0.43; 1.08) | 0.101 | 0.66 (0.42; 1.06) | 0.084 | 0.67 (0.42; 1.06) | 0.089 | 0.67 (0.42; 1.07) | 0.095 |
| Highest adherence | 24,880 / 22 | 0.63 (0.39; 1.04) | 0.072 | 0.61 (0.37; 1.01) | 0.056 | 0.62 (0.38; 1.03) | 0.065 | 0.63 (0.38; 1.04) | 0.076 |
| Trend | 121,521 / 118 | 0.86 (0.73; 1.01) | 0.064 | 0.85 (0.72; 1.00) | 0.050 | 0.85 (0.72; 1.00) | 0.057 | 0.86 (0.73; 1.01) | 0.065 |
| **HDI** |  |  |  |  |  |  |  |  |  |
| Lowest adherence | 48,725 / 47 | 1.00 (ref.) |  | 1.00 (ref.) |  | 1.00 (ref.) |  | 1.00 (ref.) |  |
| Moderately low adherence | 31,973 / 32 | 1.11 (0.72; 1.73) | 0.631 | 1.05 (0.67; 1.63) | 0.843 | 1.04 (0.67; 1.61) | 0.876 | 1.04 (0.67; 1.61) | 0.876 |
| Moderately high adherence | 23,981 / 26 | 1.19 (0.74; 1.91) | 0.469 | 1.08 (0.68; 1.74) | 0.737 | 1.08 (0.67; 1.73) | 0.763 | 1.04 (0.65; 1.68) | 0.864 |
| Highest adherence | 16,842 / 13 | 0.82 (0.45; 1.52) | 0.536 | 0.80 (0.43; 1.48) | 0.480 | 0.80 (0.43; 1.48) | 0.475 | 0.79 (0.43; 1.47) | 0.462 |
| Trend | 121,521 / 118 | 0.99 (0.84; 1.17) | 0.895 | 0.97 (0.82; 1.15) | 0.709 | 0.97 (0.81; 1.15) | 0.691 | 0.96 (0.81; 1.14) | 0.634 |

Data are presented as adjusted hazard ratios (HRs) with 95% confidence intervals (CIs). Low adherence to the dietary score (quartile 1) was used as the reference group. The trend represents the risk of dementia associated with each 1-quartile increase in adherence. Analyses were performed as follows: Model 0 was unadjusted; Model 1 was adjusted for sociodemographic factors (age, sex, education, and ethnicity); Model 2 was further adjusted for lifestyle-related factors (smoking, alcohol intake, physical activity, social interaction, and PM2.5 exposure); and Model 3 was additionally adjusted for health-related factors (BMI, hypertension, diabetes, depression, history of traumatic brain injury, hearing problems, LDL cholesterol, and cardiovascular medications).

**Table S7.** Associations of levels of adherence to Dietary Indices with Non-vascular dementia incidence.

|  |  | **Model 0** |  | **Model 1** |  | **Model 2** |  | **Model 3** |  |
| --- | --- | --- | --- | --- | --- | --- | --- | --- | --- |
| **MIND** | **Total n / cases** | **HR (95% CI)** | **p-value** | **HR (95% CI)** | **p-value** | **HR (95% CI)** | **p-value** | **HR (95% CI)** | **p-value** |
| Lowest adherence | 33,511 / 162 | 1.00 (ref.) |  | 1.00 (ref.) |  | 1.00 (ref.) |  | 1.00 (ref.) |  |
| Moderately low adherence | 42,230 / 178 | 0.86 (0.70; 1.06) | 0.170 | 0.83 (0.67; 1.02) | 0.090 | 0.85 (0.68; 1.04) | 0.127 | 0.85 (0.69; 1.04) | 0.131 |
| Moderately high adherence | 19,070 / 79 | 0.85 (0.65; 1.11) | 0.241 | 0.83 (0.63; 1.08) | 0.181 | 0.85 (0.65; 1.12) | 0.265 | 0.85 (0.65; 1.12) | 0.263 |
| Highest adherence | 26,710 / 84 | 0.68 (0.53; 0.88) | 0.004 | 0.64 (0.49; 0.83) | 0.001 | 0.67 (0.51; 0.87) | 0.003 | 0.67 (0.51; 0.87) | 0.003 |
| Trend | 121,521 / 503 | 0.89 (0.82; 0.96) | 0.005 | 0.87 (0.80; 0.95) | 0.001 | 0.88 (0.81; 0.96) | 0.005 | 0.88 (0.81; 0.96) | 0.004 |
| **MDS** |  |  |  |  |  |  |  |  |  |
| Lowest adherence | 6,556 / 28 | 1.00 (ref.) |  | 1.00 (ref.) |  | 1.00 (ref.) |  | 1.00 (ref.) |  |
| Moderately low adherence | 42,912 / 218 | 0.67 (0.57; 0.79) | <0.0001 | 0.78 (0.67; 0.92) | 0.003 | 0.77 (0.64; 0.93) | 0.008 | 0.78 (0.64; 0.93) | 0.009 |
| Moderately high adherence | 27,697 / 98 | 0.47 (0.38; 0.79) | <0.0001 | 0.54 (0.43; 0.67) | <0.0001 | 0.54 (0.43; 0.68) | <0.0001 | 0.54 (0.43; 0.68) | <0.0001 |
| Highest adherence | 44,356 / 159 | 0.48 (0.40; 0.57) | <0.0001 | 0.55 (0.46; 0.66) | <0.0001 | 0.55 (0.46; 0.67) | <0.0001 | 0.55 (0.46; 0.67) | <0.0001 |
| Trend | 121,521 / 503 | 0.76 (0.72; 0.80) | <0.0001 | 0.80 (0.75; 0.85) | <0.0001 | 0.80 (0.76; 0.85) | <0.0001 | 0.80 (0.76; 0.86) | <0.0001 |
| **RFS** |  |  |  |  |  |  |  |  |  |
| Lowest adherence | 35,375 / 151 | 1.00 (ref.) |  | 1.00 (ref.) |  | 1.00 (ref.) |  | 1.00 (ref.) |  |
| Moderately low adherence | 33,267 / 155 | 1.02 (0.82; 1.27) | 0.828 | 0.95 (0.76; 1.18) | 0.676 | 0.96 (0.77; 1.19) | 0.735 | 0.96 (0.77; 1.19) | 0.724 |
| Moderately high adherence | 27,999 / 117 | 0.93 (0.73; 1.17) | 0.550 | 0.83 (0.66; 1.06) | 0.145 | 0.84 (0.66; 1.07) | 0.169 | 0.84 (0.66; 1.07) | 0.165 |
| Highest adherence | 24,880 / 80 | 0.72 (0.55; 0.95) | 0.020 | 0.61 (0.47; 0.80) | <0.0001 | 0.61 (0.47; 0.81) | 0.001 | 0.61 (0.47; 0.80) | <0.0001 |
| Trend | 121,521 / 503 | 0.91 (0.84; 0.98) | 0.024 | 0.86 (0.79; 0.93) | <0.0001 | 0.86 (0.79; 0.93) | <0.0001 | 0.86 (0.79; 0.93) | <0.0001 |
| **HDI** |  |  |  |  |  |  |  |  |  |
| Lowest adherence | 48,725 / 190 | 1.00 (ref.) |  | 1.00 (ref.) |  | 1.00 (ref.) |  | 1.00 (ref.) |  |
| Moderately low adherence | 31,973 / 141 | 1.14 (0.92; 1.42) | 0.209 | 1.08 (0.87; 1.34) | 0.44 | 1.08 (0.87; 1.34) | 0.471 | 1.07 (0.86; 1.33) | 0.507 |
| Moderately high adherence | 23,981 / 105 | 1.12 (0.88; 1.42) | 0.341 | 1.01 (0.80; 1.29) | 0.876 | 1.01 (0.79; 1.28) | 0.925 | 1.00 (0.78; 1.27) | 0.991 |
| Highest adherence | 16,842 / 67 | 1.01 (0.77; 1.34) | 0.903 | 0.95 (0.72; 1.25) | 0.727 | 0.94 (0.71; 1.24) | 0.667 | 0.93 (0.70; 1.23) | 0.629 |
| Trend | 121,521 / 503 | 1.02 (0.94; 1.10) | 0.626 | 0.99 (0.91; 1.07) | 0.811 | 0.98 (0.90; 1.07) | 0.740 | 0.98 (0.90; 1.06) | 0.682 |

Data are presented as adjusted hazard ratios (HRs) with 95% confidence intervals (CIs). Low adherence to the dietary score (quartile 1) was used as the reference group. The trend represents the risk of dementia associated with each 1-quartile increase in adherence. Analyses were performed as follows: Model 0 was unadjusted; Model 1 was adjusted for sociodemographic factors (age, sex, education, and ethnicity); Model 2 was further adjusted for lifestyle-related factors (smoking, alcohol intake, physical activity, social interaction, and PM2.5 exposure); and Model 3 was additionally adjusted for health-related factors (BMI, hypertension, diabetes, depression, history of traumatic brain injury, hearing problems, LDL cholesterol, and cardiovascular medications).

**Table S8.** Associations of levels of adherence to Dietary Indices and Alzheimer’s Disease Incidence

|  |  | **Model 0** |  | **Model 1** |  | **Model 2** |  | **Model 3** |  |
| --- | --- | --- | --- | --- | --- | --- | --- | --- | --- |
| **MIND** | **Total n / cases** | **HR (95% CI)** | **p-value** | **HR (95% CI)** | **p-value** | **HR (95% CI)** | **p-value** | **HR (95% CI)** | **p-value** |
| Lowest adherence | 33,511 / 78 | 1.00 (ref.) |  | 1.00 (ref.) |  | 1.00 (ref.) |  | 1.00 (ref.) |  |
| Moderately low adherence | 42,230 / 91 | 0.91 (0.68; 1.23) | 0.573 | 0.90 (0.67; 1.21) | 0.508 | 0.91 (0.68; 1.22) | 0.553 | 0.90 (0.67; 1.21) | 0.524 |
| Moderately high adherence | 19,070 / 40 | 087 (0.59; 1.26) | 0.468 | 0.88 (0.60; 1.28) | 0.513 | 0.89 (0.61; 1.30) | 0.567 | 0.87 (0.59; 1.28) | 0.495 |
| Highest adherence | 26,710 / 51 | 0.83 (0.59; 1.17) | 0.292 | 0.81 (0.57; 1.15) | 0.260 | 0.83 (0.59; 1.18) | 0.315 | 0.81 (0.57; 1.15) | 0.243 |
| Trend | 121,521 / 260 | 0.94 (0.84; 104) | 0.270 | 0.93 (0.84; 1.04) | 0.260 | 0.94 (0.84; 1.05) | 0.318 | 0.93 (0.83; 1.04) | 0.240 |
| **MDS** |  |  |  |  |  |  |  |  |  |
| Lowest adherence | 6,556 / 13 | 1.00 (ref.) |  | 1.00 (ref.) |  | 1.00 (ref.) |  | 1.00 (ref.) |  |
| Moderately low adherence | 42,912 / 110 | 0.62 (0.50; 0.77) | <0.0001 | 0.73 (0.58; 0.91) | 0.006 | 0.79 (0.61; 1.02) | 0.071 | 0.78 (0.60; 1.00) | 0.058 |
| Moderately high adherence | 27,697 / 52 | 0.46 (0.34; 0.62) | <0.0001 | 0.54 (0.40; 0.72) | <0.0001 | 0.57 (0.42; 0.78) | <0.0001 | 0.56 (0.41; 0.77) | <0.0001 |
| Highest adherence | 44,356 / 85 | 0.47 (0.37; 0.60) | <0.0001 | 0.55 (0.43; 0.71) | <0.0001 | 0.58 (0.44; 0.75) | <0.0001 | 0.57 (0.44; 0.73) | <0.0001 |
| Trend | 121,521 / 260 | 0.75 (0.69; 0.81) | <0.0001 | 0.80 (0.74; 0.86) | <0.0001 | 0.82 (0.75; 0.89) | <0.0001 | 0.81 (0.75; 0.88) | <0.0001 |
| **RFS** |  |  |  |  |  |  |  |  |  |
| Lowest adherence | 35,375 / 80 | 1.00 (ref.) |  | 1.00 (ref.) |  | 1.00 (ref.) |  | 1.00 (ref.) |  |
| Moderately low adherence | 33,267 / 80 | 1.00 (0.74; 1.35) | 0.972 | 0.94 (0.69; 1.27) | 0.708 | 0.94 (0.70; 1.27) | 0.717 | 0.93 (0.69; 1.26) | 0.683 |
| Moderately high adherence | 27,999 / 57 | 0.84 (0.60; 1.17) | 0.306 | 0.76 (0.55; 1.07) | 0.121 | 0.76 (0.55; 1.07) | 0.121 | 0.76 (0.54; 1.06) | 0.109 |
| Highest adherence | 24,880 / 43 | 0.72 (0.50; 1.04) | 0.087 | 0.62 (0.43; 0.90) | 0.013 | 0.62 (0.43; 0.90) | 0.013 | 0.61 (0.42; 0.88) | 0.010 |
| Trend | 121,521 / 260 | 0.89 (0.80; 1.00) | 0.059 | 0.85 (0.76; 0.95) | 0.007 | 0.85 (0.76; 0.95) | 0.007 | 0.85 (0.76; 0.95) | 0.005 |
| **HDI** |  |  |  |  |  |  |  |  |  |
| Lowest adherence | 48,725 / 95 | 1.00 (ref.) |  | 1.00 (ref.) |  | 1.00 (ref.) |  | 1.00 (ref.) |  |
| Moderately low adherence | 31,973 / 66 | 1.07 (0.79; 1.47) | 0.627 | 1.02 (0.74; 1.39) | 0.888 | 1.01 (0.74; 1.38) | 0.926 | 1.00 (0.73; 1.37) | 0.971 |
| Moderately high adherence | 23,981 / 62 | 1.32 (0.96; 1.82) | 0.083 | 1.20 (0.87; 1.66) | 0.242 | 1.20 (0.87; 1.65) | 0.263 | 1.16 (0.84; 1.61) | 0.344 |
| Highest adherence | 16,842 / 37 | 1.13 (0.77; 1.65) | 0.508 | 1.07 (0.73; 1.57) | 0.701 | 1.05 (0.72; 1.55) | 0.767 | 1.02 (0.69; 1.50) | 0.898 |
| Trend | 121,521 / 260 | 1.07 (0.96; 1.20) | 0.194 | 1.04 (0.93; 1.17) | 0.398 | 1.04 (0.93; 1.16) | 0.452 | 1.03 (0.92; 1.15) | 0.586 |

Data are presented as adjusted hazard ratios (HRs) with 95% confidence intervals (CIs). Low adherence to the dietary score (quartile 1) was used as the reference group. The trend represents the risk of dementia associated with each 1-quartile increase in adherence. Analyses were performed as follows: Model 0 was unadjusted; Model 1 was adjusted for sociodemographic factors (age, sex, education, and ethnicity); Model 2 was further adjusted for lifestyle-related factors (smoking, alcohol intake, physical activity, social interaction, and PM2.5 exposure); and Model 3 was additionally adjusted for health-related factors (BMI, hypertension, diabetes, depression, history of traumatic brain injury, hearing problems, LDL cholesterol, and cardiovascular medications).

**Table S9.** Associations of levels of adherence to dietary indices with dementia outcomes using a 5-Year landmark analysis.

|  | **All-cause dementia** | | | **Vascular dementia** | | | | **Non-vascular dementia** | | | **Alzheimer’s Disease** | | | |
| --- | --- | --- | --- | --- | --- | --- | --- | --- | --- | --- | --- | --- | --- | --- |
| **MIND** | **Total n / cases** | **HR (95% CI)** | **p-value** | **Total n / cases** | **HR (95% CI)** | **p-value** | **Total n / cases** | | **HR (95% CI)** | **p-value** | | **Total n / cases** | **HR (95% CI)** | **p-value** |
| Lowest adherence | 33,489 / 202 | 1.00 (Ref.) |  | 33,489 / 33 | 1.00 (ref.) |  | 33,489 / 147 | | 1.00 (ref.) |  | | 33,489 / 72 | 1.00 (ref.) |  |
| Moderately low adherence | 42,212 / 210 | 0.92 (0.76; 1.12) | 0.458 | 42,212 / 46 | 1.11 (0.73; 1.69) | 0.615 | 42,212 / 164 | | 0.87 (0.70; 1.09) | 0.251 | | 42,212 / 83 | 0.88 (0.64; 1.19) | 0.423 |
| Moderately high adherence | 19,066 / 83 | 0.83 (0.64; 1.07 | 0.169 | 19,066 / 8 | 0.43 (0.20; 0.94) | 0.034 | 19,066 / 75 | | 0.91 (0.69; 1.21) | 0.541 | | 19,066 / 37 | 0.87 (0.58; 1.29) | 0.496 |
| Highest adherence | 26,698 / 92 | 0.66 (0.52; 0.85) | 0.002 | 26,698 / 15 | 0.64 (0.35; 0.97) | 0.038 | 26,698 / 77 | | 0.68 (0.51; 0.89) | 0.006 | | 26,698 / 43 | 0.87 (0.52; 1.09) | 0.140 |
| Trend | 121,465 / 565 | 0.88 (0.81; 0.95) | 0.001 | 121,465 / 102 | 0.82 (0.68; 0.98) | 0.035 | 121,465 / 463 | | 0.89 (0.82; 0.97) | 0.012 | | 121,465 / 235 | 0.91 (0.81; 1.03) | 0.152 |
| **MDS** |  |  |  |  |  |  |  | |  |  | |  |  |  |
| Lowest adherence | 6,548 / 27 | 1.00 (Ref.) |  | 6,548 / 10 | 1.00 (ref.) |  | 6,548 / 22 | | 1.00 (ref.) |  | | 6,548 / 11 | 1.00 (ref.) |  |
| Moderately low adherence | 42,890 / 246 | 0.79 (0.66; 0.93) | 0.007 | 42,890 / 38 | 0.68 (0.46; 1.00) | 0.051 | 42,890 / 203 | | 0.82 (0.68; 1.00) | 0.052 | | 42,890 / 101 | 0.78 (0.60; 1.02) | 0.076 |
| Moderately high adherence | 27,681 / 109 | 0.53 (0.43; 0.66) | <0.0001 | 27,681 / 23 | 0.51 (0.32; 0.81) | 0.005 | 27,681 / 86 | | 0.54 (0.42; 0.68) | <0.0001 | | 27,681 / 44 | 0.55 (0.60; 1.02) | <0.0001 |
| Highest adherence | 44,346 / 183 | 0.57 (0.48; 0.68) | <0.0001 | 44,346 / 31 | 0.47 (0.31; 0.71) | <0.0001 | 44,346 / 152 | | 0.60 (0.50; 0.74) | <0.0001 | | 44,346 / 79 | 0.59 (0.45; 0.77) | <0.0001 |
| Trend | 121,465 / 565 | 0.81 (0.76; 0.86) | <0.0001 | 121,465 / 102 | 0.77 (0.67; 0.87) | <0.0001 | 121,465 / 463 | | 0.82 (0.77; 0.88) | <0.0001 | | 121,465 / 235 | 0.82 (0.75; 0.89) | <0.0001 |
| **RFS** |  |  |  |  |  |  |  | |  |  | |  |  |  |
| Lowest adherence | 35,355 / 176 | 1.00 (Ref.) |  | 35,355 / 39 | 1.00 (ref.) |  | 35,355 / 137 | | 1.00 (ref.) |  | | 35,355 / 71 | 1.00 (ref.) |  |
| Moderately low adherence | 33,250 / 162 | 0.89 (0.72; 1.09) | 0.276 | 33,250 / 18 | 0.48 (0.28; 0.80) | 0.006 | 33,250 / 144 | | 1.00 (0.79; 1.26) | 0.971 | | 33,250 / 73 | 0.99 (0.72; 1.35) | 0.958 |
| Moderately high adherence | 27,985 / 130 | 0.82 (0.65; 1.03) | 0.090 | 27,985 / 25 | 0.70 (0.26; 1.14) | 0.158 | 27,985 / 105 | | 0.85 (0.66; 1.09) | 0.221 | | 27,985 / 52 | 0.79 (0.55; 1.12) | 0.192 |
| Highest adherence | 24,875 / 97 | 0.66 (0.52; 0.85) | 0.001 | 24,875 / 20 | 0.68 (0.40; 1.15) | 0.151 | 24,875 / 77 | | 0.67 (0.51; 0.89) | 0.006 | | 24,875 / 39 | 0.65 (0.50; 0.94) | 0.029 |
| Trend | 121,465 / 565 | 0.88 (0.81; 0.95) | 0.001 | 121,465 / 102 | 0.88 (0.74; 1.05) | 0.165 | 121,465 / 463 | | 0.88 (0.81; 0.96) | 0.004 | | 121,465 / 235 | 0.86 (0.77; 0.97) | 0.016 |
| **HDI** |  |  |  |  |  |  |  | |  |  | |  |  |  |
| Lowest adherence | 48,703/ 215 | 1.00 (Ref.) |  | 48,703/ 42 | 1.00 (ref.) |  | 48,703/ 173 | | 1.00 (ref.) |  | | 48,703/ 84 | 1.00 (ref.) |  |
| Moderately low adherence | 31,956 / 156 | 1.06 (0.86; 1.30) | 0.564 | 31,956 / 25 | 0.93 (0.58; 1.51) | 0.795 | 31,956 / 131 | | 1.09 (0.87; 1.36) | 0.438 | | 31,956 / 60 | 1.02 (0.73; 1.42) | 0.886 |
| Moderately high adherence | 23,969 / 119 | 1.01 (0.81; 1.27) | 0.890 | 23,969 / 23 | 1.02 (0.61; 1.69) | 0.930 | 23,969 / 96 | | 1.01 (0.79; 1.30) | 0.885 | | 23,969 / 55 | 1,21 (0.86; 1.70) | 0.251 |
| Highest adherence | 16,837 / 75 | 0.95 (0.72; 1.23) | 0.710 | 16,837 / 12 | 0.81 (0.42; 1.54) | 0.523 | 16,837 / 63 | | 0.98 (0.73; 1.31) | 0.893 | | 16,837 / 36 | 1.17 (0.79; 1.73) | 0.427 |
| Trend | 121,465 / 565 | 0.98 (0.91; 1.07) | 0.799 | 121,465 / 102 | 0.96 (0.80; 1.15) | 0.670 | 121,465 / 463 | | 0.99 (0.91; 1.08) | 0.933 | | 121,465 / 235 | 1.07 (0.95; 1.20) | 0.254 |

Data are presented as adjusted hazard ratios (HRs) with 95% confidence intervals (CIs) per quartile of dietary patterns. Analyses were adjusted for sociodemographic factors (age, sex, education, and ethnicity), lifestyle-related factors (smoking, alcohol intake, physical activity, social interaction, and PM2.5 exposure), and health-related factors (BMI, hypertension, diabetes, depression, history of traumatic brain injury, hearing problems, LDL cholesterol and cardiovascular medications). A 5-year landmark analysis was applied.
